# Splice modulation of *COL4A5* reinstates collagen IV assembly in an organoid model of Alport syndrome

**DOI:** 10.1172/jci.insight.194759

**Published:** 2025-12-18

**Authors:** Hassan Saei, Bruno Estebe, Nicolas Goudin, Mahsa Esmailpour, Julie Haure, Olivier Gribouval, Christelle Arrondel, Vincent Moriniere, Pinyuan Tian, Rachel Lennon, Corinne Antignac, Geraldine Mollet, Guillaume Dorval

**Affiliations:** 1 Laboratory of Hereditary Kidney Diseases, INSERM UMR 1163, Imagine Institute, Université Paris Cité, Paris, France.; 2 Necker Bioimage Analysis Core Facility of the Structure Fédérative de Recherche Necker, INSERM US24/CNRS UAR 3633, Paris, France.; 3 Department of Genomic Medicine for Rare Diseases, Necker–Enfants Malades Hospital, Assistance publique, Hôpitaux de Paris (AP-HP), Paris, France.; 4 Wellcome Trust Centre for Cell-Matrix Research, University of Manchester, Manchester, United Kingdom.; 5 Department of Paediatric Nephrology, Royal Manchester Children’s Hospital, Manchester University Hospitals NHS Foundation Trust, Manchester, United Kingdom.

**Keywords:** Genetics, Nephrology, Chronic kidney disease, Collagens, Therapeutics

## Abstract

Kidney organoids are an emerging tool for disease modeling, especially genetic diseases. Among these diseases, X-linked Alport syndrome (XLAS) is a hematuric nephropathy affecting the glomerular basement membrane (GBM) secondary to pathogenic variations in the *COL4A5* gene encoding the α5 subunit of type IV collagen [α5(IV)]. In patients carrying pathogenic variations affecting splicing, the use of antisense oligonucleotides (ASOs) offers immense therapeutic hope. In this study, we develop a framework combining the use of patient-derived cells and kidney organoids to provide evidence of the therapeutic efficacy of ASOs in XLAS patients. Using multiomics analysis, we describe the development of GBM in WT and mutated human kidney organoids. We show that GBM maturation is a dynamic process, which requires long organoid culture. Then, using semi-automated quantification of α5(IV) at basement membranes in organoids carrying the splicing variants identified in patients, we demonstrate the efficacy of ASO treatment for α5(IV) restoration. These data contribute to our understanding of the development of GBM in kidney organoids and pave the way for a therapeutic screening platform for patients.

## Abstract 2

Antisense oligonucleotide therapy corrected gene splicing in kidney organoid models of Alport syndrome, advancing ASO-based strategies for treating inherited kidney diseases

## Introduction

Alport syndrome (AS) is the most frequent hereditary glomerulonephritis, due to a structural defect of the glomerular basement membrane (GBM) (1, 2). In the kidney, the main component of the basement membrane (BM) is type IV collagen, which functions as a heterotrimer of α1α2α1(IV) chains predominantly in the early nephrons, α3α4α5(IV) mainly in the mature GBM but also in distal tubules, and α5α5α6(IV) in the Bowman’s capsule and distal tubule BM (2–5). The developmental switches in the GBM of laminin from α1β1γ1 to α5β2γ1 and of type IV collagen from α1α2α1(IV) to α3α4α5(IV) networks are crucial for maintaining the integrity of the glomerular filtration barrier (2, 6). The unique property of the collagen networks in BMs is the requirement for all specific chains of type IV collagen to be present in order to synthesize heterotrimers. If one chain is absent, the remaining intact chains are unable to form trimers, and none of the trimer chains are expressed in the BM. The molecular network of these switches is not yet well understood (1).

AS is secondary to a defect in α3α4α5(IV) heterotrimers. Pathogenic variations leading to AS may involve the *COL4A3*, *COL4A4*, and *COL4A5* genes, with autosomal recessive, dominant (*COL4A3* and *COL4A4*), or X-linked (*COL4A5*) inheritance (7, 8). A relative genotype-phenotype correlation has been described, ranging from lifelong isolated hematuria to development of chronic kidney disease leading to kidney failure in early adulthood in the most severe forms (mainly autosomal recessive or X-linked in males) (9–11). To date, the renin-angiotensin system blockers are the standard of care for all forms of AS, but they only delay kidney failure, and no curative therapy exists to date.

We recently identified and validated splice-altering variations in patients with X-linked AS (XLAS) phenotype (12). In this study, we detected 14 pseudo-exon retention events in 19 patients with XLAS. These variants are perfect targets of steric-block antisense oligonucleotides (ASOs), an emerging personalized therapy that can ideally mask the newly created splice sites or splicing modulator sites and restore WT RNA transcripts and normal protein expression. However, the lack of robust in vitro models is a limitation for preclinical evaluation of ASO capability to amend splicing.

Different studies have reported the generation of various XLAS mouse models, such as *col4a5^del-ATGG^*, *col4a5^tm1Yseq^* (p.G5X), and *col4a5^–/y^* (p.Arg471*) mice (13–15). These models can replicate phenotypic characteristics comparable to those observed in humans, such as GBM abnormalities, proteinuria, and renal failure. While using mouse models offers unique benefits, such as the ability to measure renal function, the validation of therapeutic approaches remains challenging owing to the extensive time necessary for model generation and various technical limitations. For instance, intronic sequences are not conserved between humans and mice. Therefore, human intronic variants may not be easily introduced into a mouse model or faithfully reproduce the splicing defects observed in humans. As a result, it is necessary to develop more complex in vitro models that can rapidly mimic disease conditions and reflect the genetic background of the patients.

The emergence of induced pluripotent stem cell (iPSC) technology offers unprecedented insights into the previously inaccessible window for human organ development ex utero (16–18). The development of kidney organoids enables the study of kidney tissue organization and cell differentiation profiles over time (19, 20), as well as the development of kidney disease models (21–23). Although the differentiation of kidney organoids from iPSCs has evolved rapidly, it still requires optimization of the protocol to meet specific disease characteristics (16, 18, 24, 25).

In this study, we established a two-step preclinical platform to evaluate the efficacy of ASOs targeting splicing defects. In the first step, we assessed ASO activity in patient-derived primary cells carrying deep-intronic *COL4A5* variants. In the second step, we characterized iPSC-derived kidney organoids at multiple time points to refine both cellular and extracellular matrix development and to restore type IV collagen network in the BMs produced by podocytes and other *COL4A5*-expressing cells. Using confocal imaging and a multiomics approach, we thoroughly characterized organoid maturation, with a particular focus on GBM collagen composition. This analysis enabled the development of XLAS organoid models carrying various deep-intronic variants that recapitulate BM defects and allow ASO screening. This strategy provided a scalable and relevant model for in vitro ASO efficacy testing, paving the way for the development of precision therapies for patients with XLAS.

## Results

### Multiomics analysis of kidney organoids in prolonged culture reveals podocyte and ECM dynamics.

**Figure 1:**
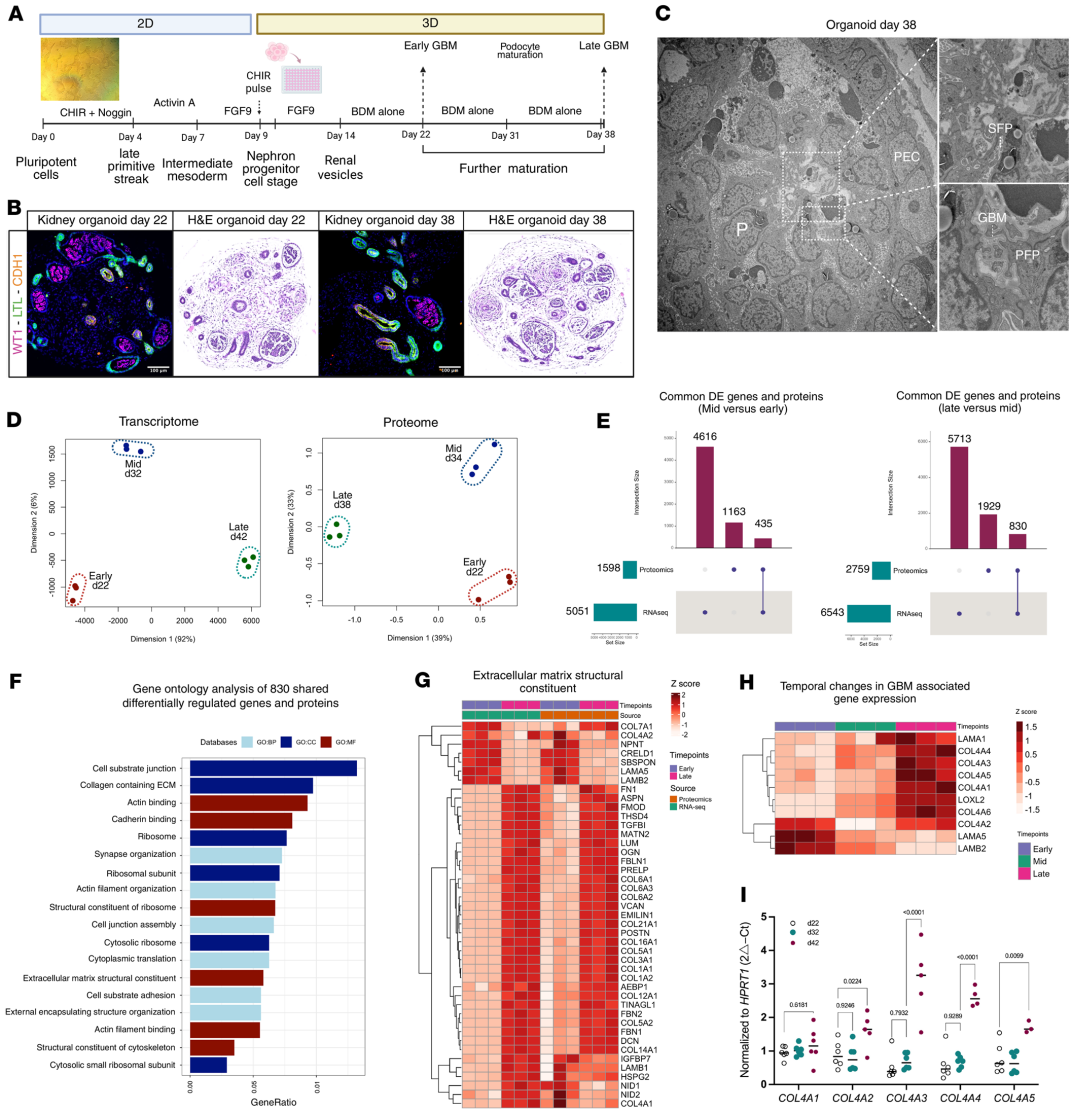
Multiomics characterization of kidney organoids in prolonged culture. (**A**) Kidney organoid differentiation protocol transitioning from 2D to 3D cultures, with focus on prolonged culture. (**B**) Immunofluorescence and hematoxylin and eosin (H&E) staining of kidney organoids at day 22 and day 38, demonstrating their structural integrity. (**C**) Transmission electron microscopy image of an organoid (day 38), highlighting glomerular structures with podocytes (P), primary and secondary-like foot processes (PFP and SFP), glomerular basement membrane (GBM), and parietal epithelial-like cells (PEC) (original magnifications: left image, ×1,250; top image, ×4,300; bottom image, ×8,500). (**D**) Multidimensional scaling plot for transcriptomic and proteome data, showing distinct clustering of samples from each time point (early, mid, and late). (**E**) Cross-matching of RNA and protein data (left: mid versus early; right: late versus mid) identified 830 shared differentially regulated biomolecules (genes and proteins) comparing late versus mid groups. (**F**) Gene Ontology enrichment analysis (GO:CC, cellular components; GO:BP, biological processes; GO:MF, molecular function) of 830 shared differentially regulated genes (fold change, 1.2) and proteins (FDR, 0.05) obtained from late versus mid culture comparison. (**G**) Heatmap visualization of differentially expressed ECM structural genes and proteins over time comparing early and late. (**H**) Heatmap of type IV collagen and laminin gene expression from bulk RNA-seq. (**I**) RT-qPCR quantification of different chains of type IV collagen of kidney organoids harvested at early, mid, and late culture, confirming GBM maturation and dynamics in prolonged culture (2-way ANOVA with Tukey’s multiple-comparison test).

Our initial goal before developing the disease model was to optimize the conditions for accurately assessing the AS molecular phenotype. We maintained organoids in culture for 38 days and beyond to evaluate BM and podocyte molecular dynamics (classical protocols terminate at day 22 of organoid culture) (Figure 1A). The integrity of the organoid differentiation was assessed by immunostaining for podocyte marker WT1, proximal tubule epithelial cell marker LTL, and distal tubule epithelial cell marker CDH1 at days 22 and 38 (Figure 1B). We checked BM ultrastructure by transmission electron microscopy on isogenic controls. We unsurprisingly did not observe the trilaminar BM, which is produced by endothelial cells and podocytes in the GBM, but nevertheless observed short segments of bilayered BM (Figure 1C). To further characterize the organoids, we performed immunostaining for endothelial cells (CD31^+^), fibroblasts (PDGFRβ^+^), myofibroblasts (ACTA2^+^), proliferating cells (HMGB2^+^), and mesangial progenitors (HOPX^+^) at day 38. Only a few endothelial cells were detected, and these were not localized within glomerular structures, nor did they form vascular networks. Fibroblasts and myofibroblasts were predominantly observed surrounding CDH1^+^ tubular epithelial structures. Proliferating cells were distributed across multiple regions, including the stromal compartment (expressing matrix protein COL1A1^+^) and subsets of tubular epithelial cells. HOPX^+^ mesangial-like cells were detected in low numbers and were not positioned within glomerular structures, indicating the absence of properly organized mesangial cells (Supplemental Figure 1, A–E; supplemental material available online with this article; https://doi.org/10.1172/jci.insight.194759DS1). Multiomics (RNA sequencing [RNA-seq] and proteomics) investigations on organoids at early (day 22), mid (day 32/34), and late (day 38/42) time points revealed dynamic changes in podocyte- and ECM-associated genes and proteins (Supplemental Figure 2, A and B). The complete list of the differentially expressed genes (DEGs) obtained from bulk RNA-seq analysis of organoids from day 32 versus day 22 and day 42 versus day 22 is provided in Supplemental Tables 1 and 2, respectively. The list of proteins differentially regulated comparing late and early time points is provided in Supplemental Tables 3 and 4. A multidimensional scaling plot on bulk RNA-seq and proteomics data revealed a clear separation of samples from different time points and their replicates, indicating substantial differences in gene and protein expression in organoids cultured for extended periods (Figure 1D). We cross-matched transcriptome and proteome data (cellular fraction merged with ECM fraction) comparing mid versus early and late versus mid time points and identified 830 shared differentially regulated biomolecules (genes and proteins) (Figure 1E). Gene set over-representation ontology analysis (top 6 enrichments in each category; see Figure 1F) revealed changes in genes and proteins associated with the extracellular matrix (ECM) component and regulation (e.g., *FBN1*, *FBN2*, *VCAN*, *COL1A1*, *COL1A2*, *COL4A1*, *LAMB2*, and *LAMA5*; Figure 1G), cell junction assembly (e.g., *TJP1*, *CLDN3*, *CLDN11*, *ITGA2*, and *CDH11*), and podocyte development (e.g., *NPHS1*, *NPHS2*, *PODXL*, *ITGA3*, and *PTPRO*; Supplemental Figure 2, C and D). Notably, based on transcriptome data, the expression of *COL4A1*, *COL4A3*, *COL4A4*, *COL4A5*, *LAMA1*, and *LOXL2* was increased at late time points, while *LAMB2* and *LAMA5* expression was significantly decreased (Figure 1H). Type IV collagen expression was further studied by quantitative PCR analysis of whole organoids harvested on days 22, 32, and 42, which confirmed the increased expression of the GBM type IV collagen network genes over time (Figure 1I). Proteomics analysis was not successful in detecting collagen α3(IV)– and α4(IV)–specific peptides, probably because of their minimal abundance compared with other type IV collagens; therefore it is not illustrated on the heatmap. Moreover, lysyl oxidase–like 2 (*LOXL2*) was significantly upregulated at late organoid time points (mRNA, 4.9-fold; protein, 1.7-fold). This increase overlapped with temporal remodeling of collagen IV isoforms, consistent with LOXL2’s role in collagen IV cross-linking (Supplemental Tables 1–4). However, we should note that *LOXL2* is not specifically expressed in podocytes; it is expressed in nearly all cell types, with particularly high expression in the stromal population. Despite challenges in detecting collagen α3(IV) and α4(IV) peptides, these findings provide valuable insights into ECM composition dynamics over time, highlighting the unique molecular adaptations within long-term cultured organoids.

**Figure 2:**
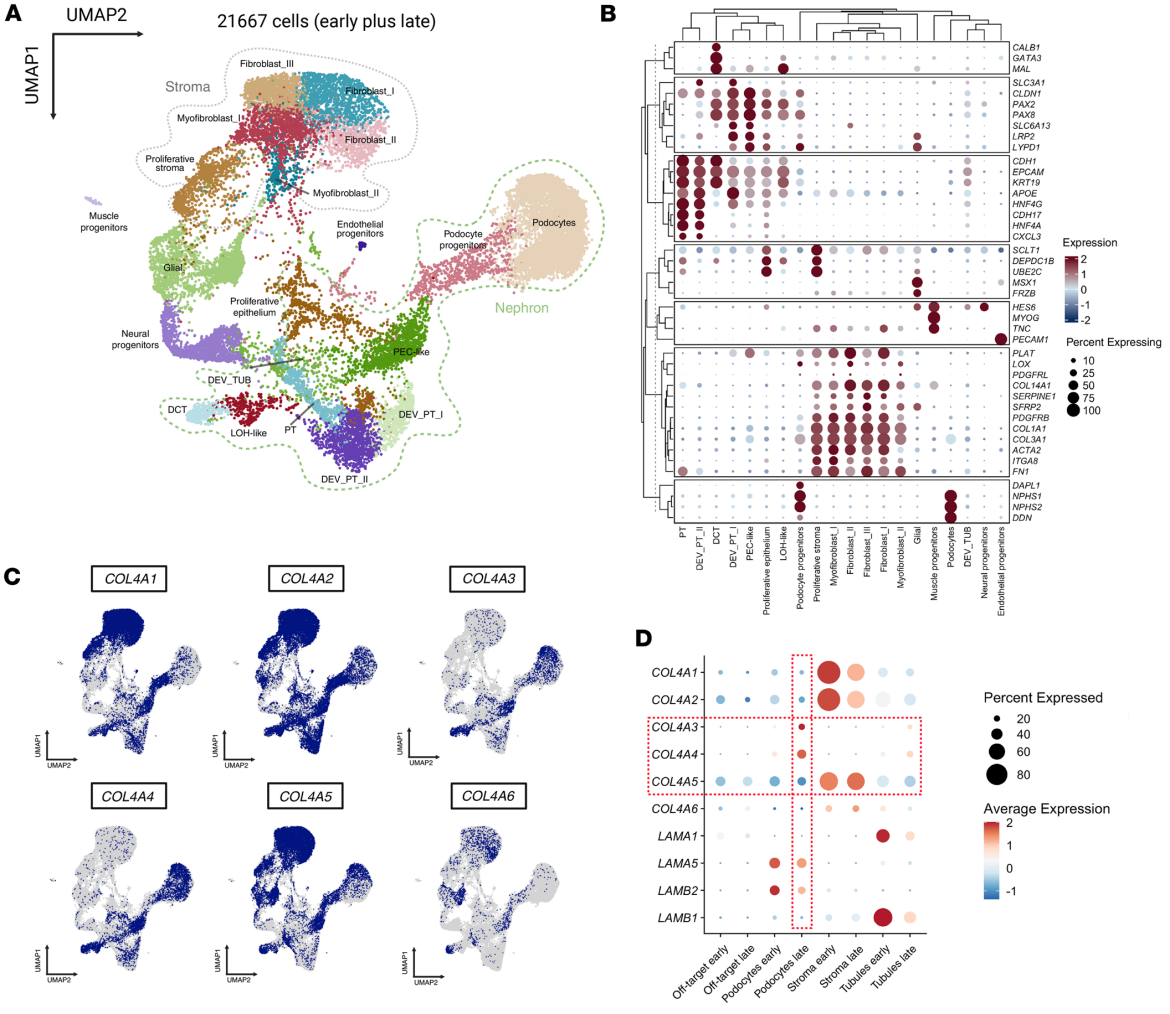
Temporal single-cell transcriptomic analysis of kidney organoid development. (**A**) UMAP visualization of integrated single-cell RNA-seq data at day 22 and day 38, identifying distinct cell populations, including nephron population, stromal population, and others (off-target population). (**B**) DotPlot representation of gene markers used to identify different cell types in the integrated dataset. (**C**) FeaturePlots representation of different cell types expressing type IV collagen chains. (**D**) Dot plots of collagen type IV and laminin gene family expression across cell types and time points in the single-cell dataset.

By performing single-cell RNA-seq on kidney organoids at days 22 and 38 (21,667 cells after integration), we aimed to verify which cell types were contributing more to the changes in ECM gene expression. We confirmed the existence of 3 main clusters: nephron, stromal, and off-target clusters (Figure 2A). Marker genes used to annotate each cell type are presented in Figure 2B and Supplemental Table 5. Focusing on podocyte populations, we observed different clusters including podocyte progenitors (expressing *DAPL1* and *LYPD1*) and differentiated podocytes (expressing *DDN*). This indicated the presence of distinct podocyte populations within the organoids, each at different developmental stages. We also visualized, using FeaturePlot, which cell types express the different collagen IV chains (Figure 2C). Gene expression analysis comparing 2 time points, with a focus on the 3 main clusters, revealed an upregulation of GBM type IV collagen genes supporting GBM maturation and type IV collagen upregulation by day 38. Analysis of the podocyte cluster in our dataset revealed a marked increase in *COL4A3* and *COL4A4* expression by day 38, whereas *COL4A1* and *COL4A2* expression decreased in stromal cells (Figure 2D). No significant difference in *COL4A5* expression was observed in podocytes when day 22 was compared with day 38. A slight decrease in *LAMB2* and *LAMA5* gene expression was also noted in podocytes at day 38, as shown by global transcriptome and proteome analyses.

**Figure 3:**
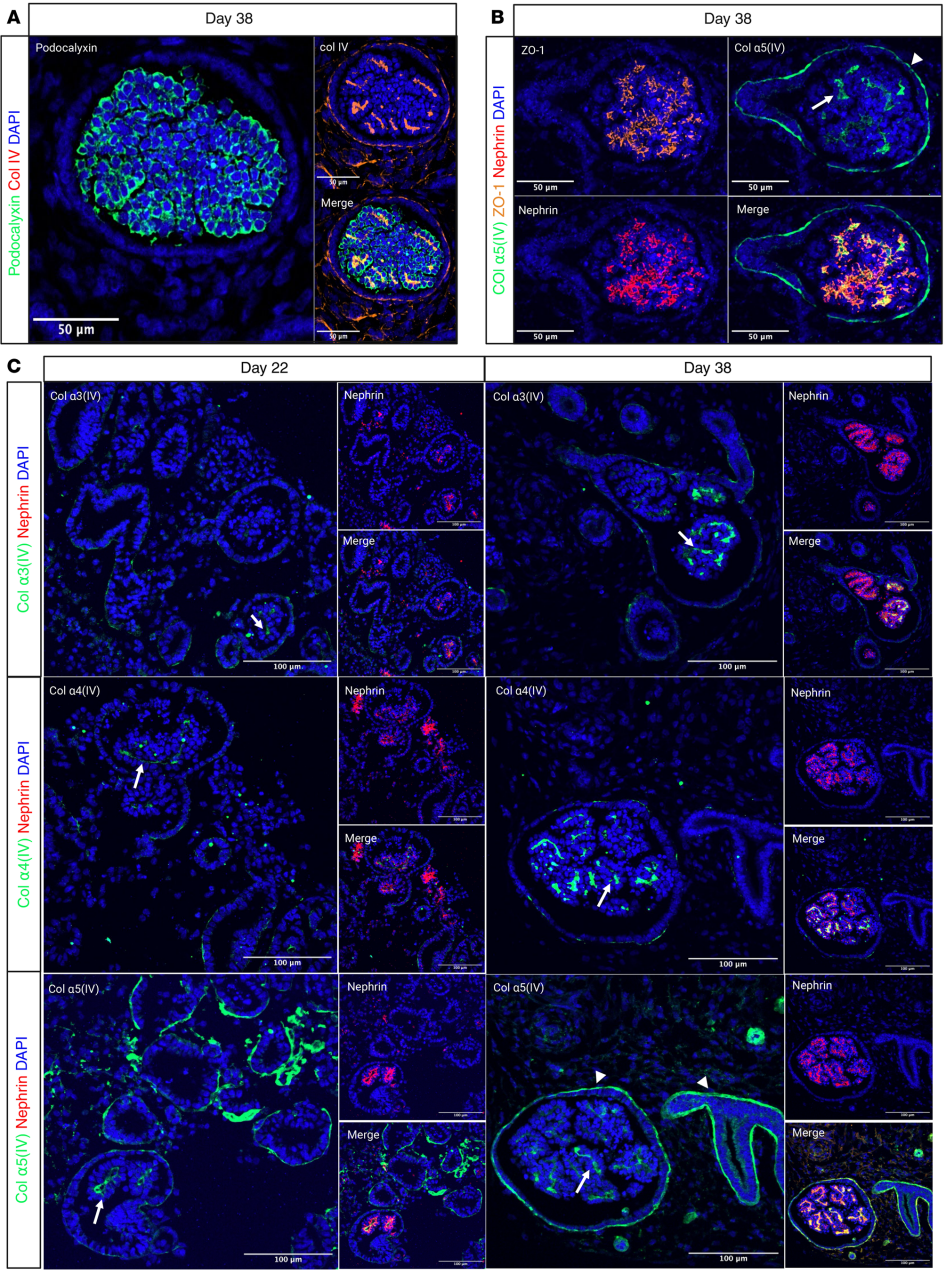
Immunofluorescence staining of kidney organoids highlighting the localization of various junctional and ECM-associated proteins relevant to podocyte maturation. (**A**) Spatial distribution of podocalyxin (in green) and type IV collagen (in red) in organoid (day 38 of culture). Podocalyxin is mainly localized at the apical side of the podocytes, while type IV collagen (captures all chains α1–α6) is present at the basal side, forming the GBM. Scale bars: 50 μm. (**B**) Colocalization of tight junction protein (ZO-1) (in orange), nephrin (in red), and collagen α5(IV) (in green), at the basal side. Scale bars: 50 μm. (**C**) Type IV collagen network localization and abundance at days 22 and 38 of culture. This panel confirms the increased production of collagen α3(IV) and α4(IV) chains at day 38 and their deposition in the GBM (white arrows point to α3α4α5 and arrowheads point to α5α6α5 heterotrimers in the PEC-BM and TBM). Scale bars: 100 μm.

We performed further organoid characterization through immunostaining for various proteins. Since we did not find the collagen α5(IV) antibodies to be specific, we inserted a V5 tag in exon 2 of the *COL4A5* gene, following the signal peptide. Therefore, labeling of the α5(IV) collagen in this study was always performed using anti–V5 tag antibody. By colabeling podocalyxin with type IV collagen, and tight junction protein 1 (ZO-1) with collagen α5(IV), we confirmed podocyte apical and basal polarity (Figure 3, A and B). In the mature kidney, collagen α3(IV), α4(IV), and α5(IV) chains are produced by the podocyte and also by the distal tubular cells. The α5(IV) chain is also produced by the parietal epithelial-like cells (PECs). Here, labeling of α3(IV), α4(IV), and α5(IV) collagens on days 22 and 38 revealed minimal deposition of the GBM collagen network, α3α4α5(IV), on day 22, with increased production and secretion of this network by day 38 (Figure 3C). In addition, as in the kidney, we observed expression of the α5(IV) chain lining the cells surrounding the podocytes, which we identified as PEC-like cells, based on positive claudin-1 staining (Supplemental Figure 3A). Further BM analysis using laminin-specific antibodies (anti–laminin-β1, –laminin-β2, and –laminin-α5) at different time points confirmed the establishment of a mature laminin network as early as day 22, which was expected since the laminin switch occurs earlier in the development than the type IV collagen switch (26) (Supplemental Figure 3B). Therefore, prolonged organoid culture is necessary for ECM maturation, particularly for the type IV collagen switch in the GBM, which is a key factor in the development of AS organoid models.

### XLAS organoid model with deep-intronic variations recapitulates BM defects.

**Figure 4:**
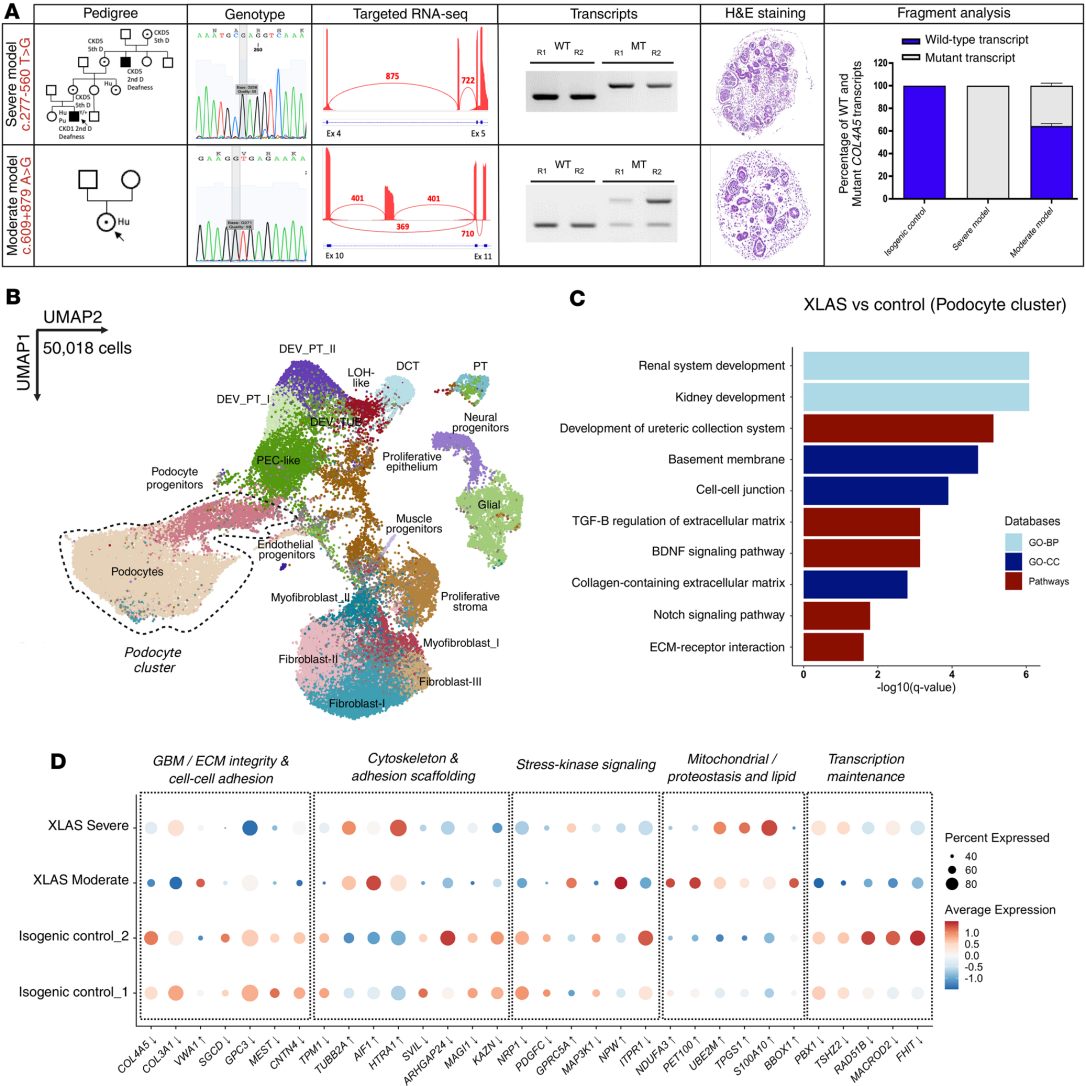
Characterization of XLAS organoid models with deep-intronic variations. (**A**) Pedigrees of patients and genotypes of the severe and moderate variants introduced in a control male iPSC line. Multilevel characterization of the molecular phenotype in organoid models using Sanger sequencing, targeted RNA-seq, RT-PCR results (R1 and R2 indicate repeats 1 and 2), H&E staining of XLAS organoids, and fragment analysis. (**B**) UMAP plot depicting 50,018 cells integrated in various cell types, including distinct podocyte clusters. (**C**) Gene Ontology analysis comparing mutant (severe and moderate) versus control podocytes, showing enriched kidney- and ECM-related pathways. (**D**) Dot plot showing expression of top up- and downregulated genes obtained from comparing diseased podocytes and controls, with dot size indicating percentage expressed and color intensity representing average expression. Functional labels were assigned based on curated roles. The differences observed between isogenic controls 1 and 2 reflect transcriptional variations between differentiation batches.

For preclinical assessment of ASO therapy, we generated two XLAS kidney organoid models by introducing deep-intronic variations in the *COL4A5* gene in the previously used control male iPSC line containing the V5 tag in the *COL5A5* gene. Both variations (c.277–560T>G identified in patient P3 and c.609+879A>G identified in patient P14) lead to the creation of a new splice site and to intron retention (IR) but with a complete absence of the WT transcript for the former event and residual expression of the WT transcript for the latter, which were thus expected to lead to a severe and a moderate model, respectively (12) (Figure 4). Targeted RNA-seq and fragment analysis confirmed aberrant *COL4A5* splicing in both XLAS organoid models. Hematoxylin and eosin staining revealed no phenotypic differences in differentiation between the models (Figure 4A).

Single-cell RNA-seq was performed on severe and moderate XLAS models on day 22 to determine the molecular aspects of the disease at an early time point (Figure 4B). Four single-cell experiment datasets were integrated (50,018 cells), and cell types were annotated using knowledge-based approaches and previously defined marker genes (Figure 2B). To identify differentially regulated genes in this experiment, we combined the 2 mutants into a single group, and performed differential expression testing using the DESeq2 method. This approach resulted in the identification of 391 DEGs (39 upregulated and 352 downregulated) (Supplemental Table 6). The over-representation analysis of DEGs identified multiple pathways and ontology terms, including renal system/kidney development highlighting kidney-specific gene dysregulation. Moreover, the analysis revealed dysregulation of BM, ECM, and cell-cell junction in diseased podocytes, confirming the disease molecular pathology (Figure 4C). A complete list of genes enriched in each gene set library is shown in Supplemental Table 7. Podocytes in XLAS organoids showed reduced expression of key GBM/ECM components (*COL4A5*, *COL3A1*, *SGCD*, *CNTN4*), indicating impaired matrix integrity, with *VWA1* as a possible compensatory response. Notable shifts in the expression of genes associated with cytoskeleton and cell-cell adhesion (*TPM1*, *SVIL*, *ARHGAP24*) were observed toward injury or remodeling (*HTRA1*, *AIF1*, *TUBB2A*). Signaling molecules linked to PDGF/VEGF/MAPK pathways (*PDGFC*, *NRP1*, *MAP3K1*) were downregulated, while alternative stress– and GPCR-related signals (*GPRC5A*, *NPW*) were upregulated. In contrast, mitochondrial and proteostasis-associated genes (*NDUFA3*, *PET100*, *UBE2M*, *TPGS1*) were induced, consistent with probable metabolic adaptation. Together, these findings suggest that podocytes respond to *COL4A5* deficiency with ECM loss, cytoskeletal remodeling, induction of pathways associated with stress and injury, and metabolic compensation (Figure 4D). In addition to differential gene expression analysis, we performed in silico analysis of apoptosis using UCell (27) and a list of marker genes (*CASP3*, *CASP7*, *CASP8*, *CASP9*, *BAX*, *BAK1*, *BID*, *BAD*, *TP53*, *APAF1*, *CYCS*, *PMAIP1*, *BBC3*). No increase in apoptosis signature was detected in XLAS organoids compared with controls (mean value of the calculated score for isogenic controls = 0.0108, for severe model = 0.0126, and for moderate model = 0.0124). To evaluate the relevance of the small differences observed, we calculated the effect size using Cliff’s delta (28), which fell within the negligible range (<0.147), indicating that the differences are not biologically meaningful. Overall, the single-cell RNA-seq experiment confirmed the enrichment of disease-associated pathways within the podocyte cluster, further supporting the relevance of organoids for XLAS modeling.

**Figure 5:**
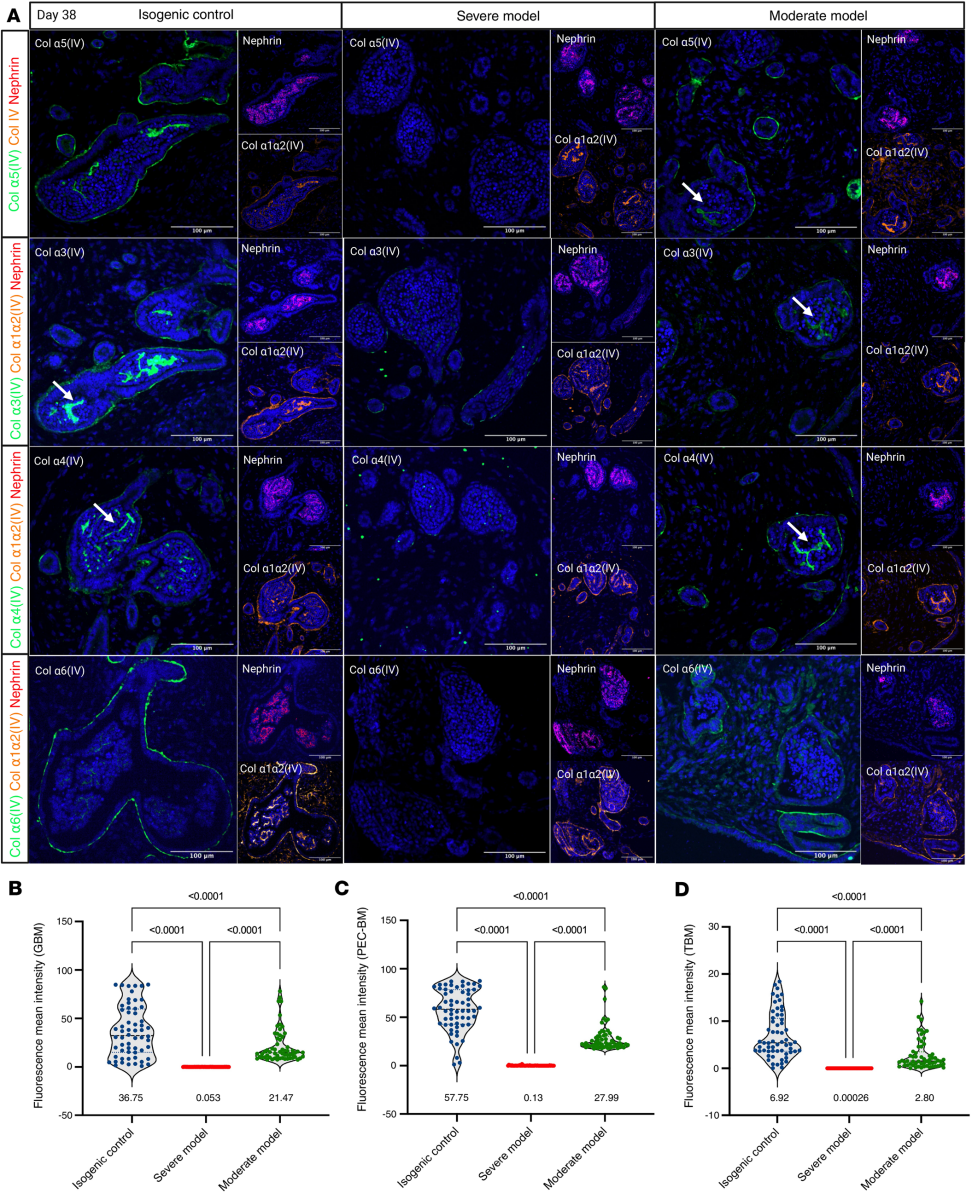
Analysis of BM defects in XLAS. (**A**) Immunofluorescence staining for collagen α3(IV), α4(IV), α5(IV), and α6(IV) chains in organoids (day 38), showing the absence of type IV collagen networks (both α5α5α6 and α3α4α5) in the severe XLAS model and substantial decrease in the moderate XLAS model. White arrows point to the GBM. Scale bars: 100 μm. (**B**–**D**) Quantification of collagen α5(IV) mean fluorescence intensities within GBM (**B**), PEC-BM (**C**), and TBM (**D**) in the severe, moderate, and control organoids (*n* = 60 objects in each group; each object is either tubule or glomerulus) (values below each condition indicate the mean intensity, 1-way ANOVA with Tukey’s multiple-comparison test). No collagen α5(IV) secretion was observed in the XLAS severe models, and significant reduction in collagen α5(IV) deposition was observed in all BMs in the moderate model.

Immunofluorescence staining of organoids at day 38 using type IV collagen chain-specific antibodies confirmed as expected the absence of collagen α3(IV), α4(IV), α5(IV), and α6(IV) proteins in the GBM, tubular basement membrane (TBM), and PEC-BM in the severe model and their qualitatively diminished expression in the moderate model (Figure 5A). To ensure reliable interpretation after therapy development, we developed a macro for Fiji (see Methods) to semi-automate the quantification of BM proteins and perform a comparative analysis (Supplemental Figure 4). The mean fluorescence intensity of collagen α5(IV) in the GBM was 0.053 in the severe model, 21.47 in the moderate model, and 36.75 in the control (Figure 5B). In the PEC-BM, the intensity was 0.13, 27.99, and 57.75, respectively, and for the TBM, the values were 0.00026, 2.80, and 6.92, respectively (Figure 5, C and D). This macro enabled accurate and rapid quantification of collagen α5(IV) in different BMs in the diseased and isogenic control organoids. Quantification of collagen α5(IV) mean fluorescence intensity revealed the absence of type IV collagen deposition in various BMs in the severe model. It also confirmed a significant decrease in signal intensity, indicating the defect in the moderate model.

### Splicing events in patient-derived primary cells and kidney organoids show amenability to ASO therapy.

**Figure 6:**
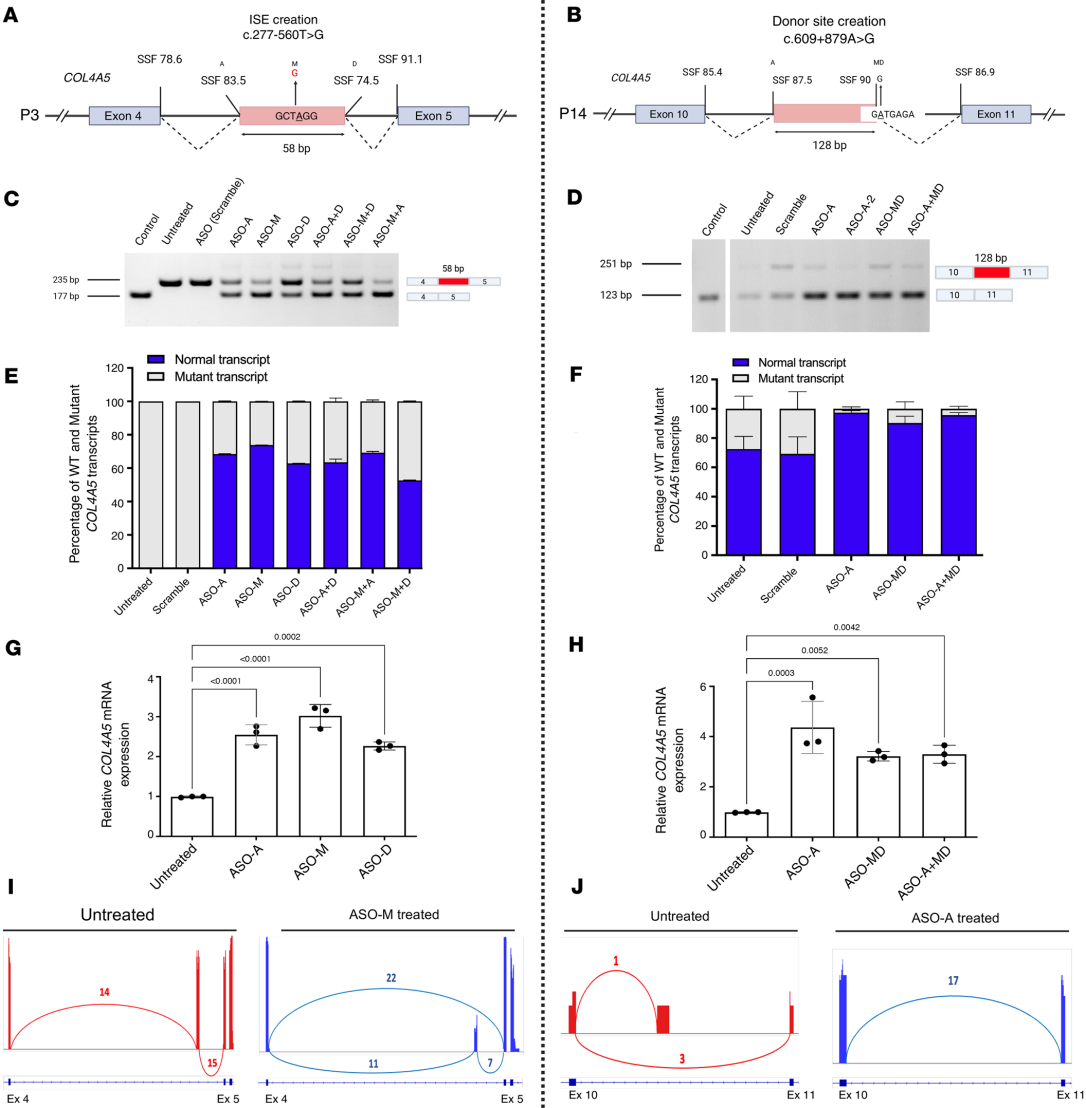
Evaluation of ASO efficacy in correcting splicing defects in patient-derived fibroblasts with deep-intronic *COL4A5* variants. (**A** and **B**) Schematic representation of the *COL4A5* variants identified in patients P3 (**A**) (c.277+560T>G) and P14 (**B**) (c.609+879A>G), which create an intronic splice enhancer (ISE) and new donor splice site, respectively, leading to intron retention in both cases. Splice Site Finder (SSF) (Alamut Visual Plus software) score for each site is shown. (**C** and **D**) RT-PCR analysis of ASO-treated and untreated fibroblasts, where ASO treatments reduced the levels of the mutant transcript and increased the proportion of the WT transcript. In P14, 2 faint bands are observed, which are due to the degradation of mutant mRNA mediated by nonsense-mediated decay, and to a low residual WT production. (**E** and **F**) Quantification of the relative expression of WT and mutant transcripts across treatments by fragment analysis (*n* = 3, 2-way ANOVA), showing the increase of WT/mutant transcript ratio after ASO treatment. (**G** and **H**) RT-qPCR quantification of *COL4A5* mRNA normalized to *HPRT1* shows a significant increase after ASO treatments with *P* value indicated above each bar plot (*n* = 3, ANOVA with Tukey’s multiple-comparison test). (**I** and **J**) Sashimi plots for untreated and ASO-treated samples, showing changes in splice junction usage before and after treatment.

We used a two-step strategy for ASO therapy development. As a first step we decided to validate the ASO-mediated restoration of *COL4A5* splicing in patient-derived fibroblasts. For this purpose, we selected 6 IR events observed in 7 independent patients (12), P3, P10, P14, P15/P20, P16, and P19 (Table 1). Up to 3 ASOs were designed (see Methods) to correct each IR event in mature *COL4A5* mRNA (the sequences and details of all ASOs are described in Table 1). We corrected aberrant splicing in all (7/7) tested patient fibroblasts with deep-intronic variations in the *COL4A5* gene (for P3 and P14 see Figure 6, A and B, and for P10, P15/P20, P16, and P19 see Supplemental Figure 5) using Lipofectamine-based ASO transfection. In particular, when testing 3 ASOs and their combination in fibroblasts of patients P3 and P14, we observed the best substantial increase in the WT/mutant transcript ratio with ASO-M (0% in untreated cells to 73.9% in treated cells) and with ASO-A (69.26% in untreated cells to 96.46% in treated cells), in fibroblasts from patients P3 and P14, respectively (Figure 6, C–F). These results were confirmed by quantitative reverse transcription PCR (RT-qPCR) (Figure 6, G and H) and targeted RNA-seq (Figure 6, I and J). This ASO screening in patient fibroblasts allowed us to select the best ASOs to be used in the second step of our ASO screening framework using XLAS organoids.

**Figure 7:**
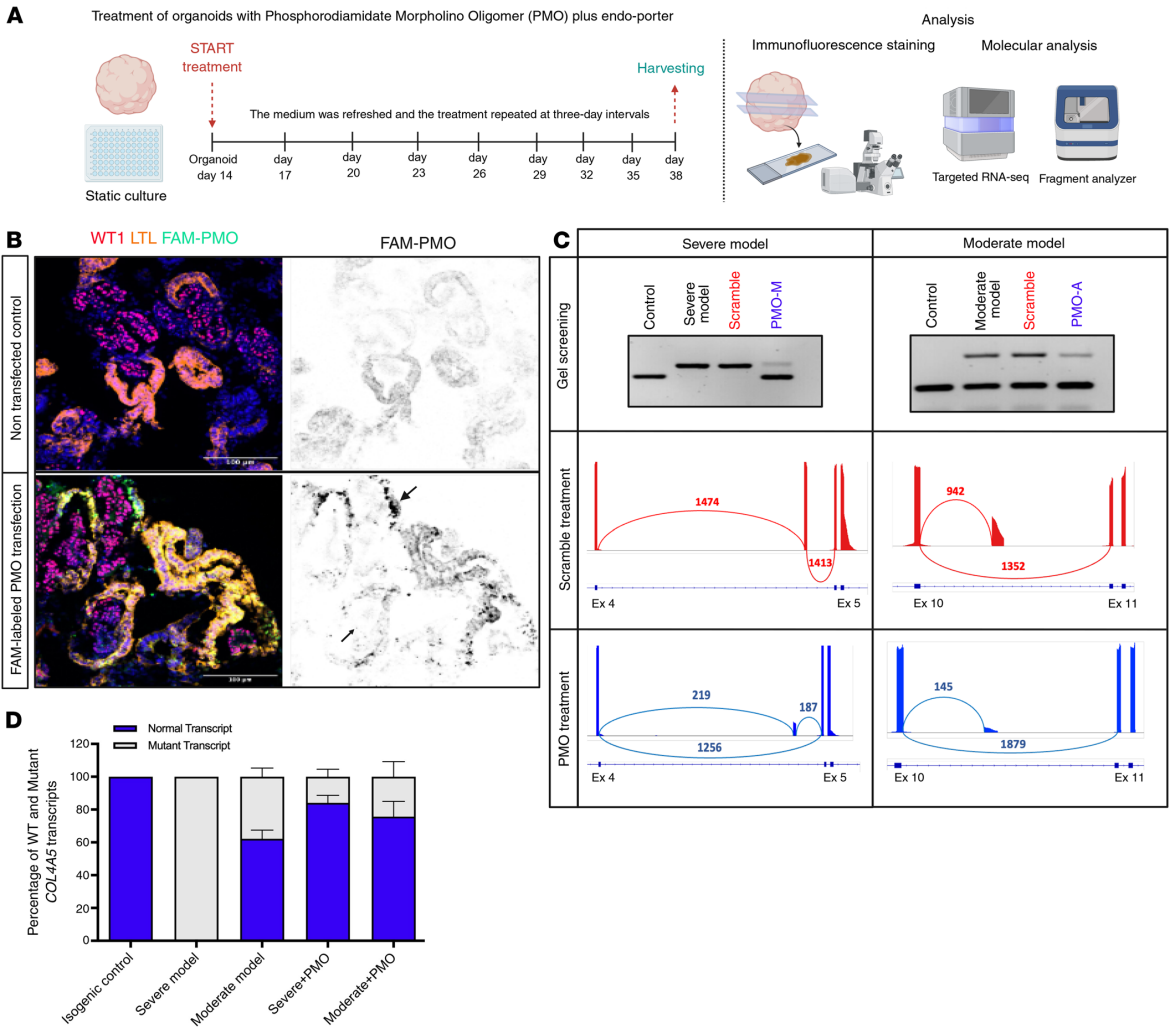
Successful restoration of aberrant splicing in XLAS organoid models by antisense oligonucleotide therapy. (**A**) Experimental timeline of the PMO treatment (5 μM) in organoids, starting at day 10+4, including media changes every 3 days and harvesting for molecular imaging–based analysis at day 38. (**B**) Immunofluorescence images showing PMO uptake (FAM-PMO, green) in PEC-like cells and tubule LTL^+^ cells (yellow) and podocytes after a single PMO transfection over 3 days (started at 10+8), demonstrating efficient uptake in tubular epithelial cells, PEC-like cells, and podocytes (black arrows). Scale bar: 100 µm. (**C**) RT-PCR and targeted RNA-seq results before and after PMO treatment of both severe and moderate models showing a reduction of mutant *COL4A5* transcript and correction of splicing following PMO treatment in both models. (**D**) Quantification of normal to mutant *COL4A5* transcript ratio, demonstrating a promising splice modulation capability of PMOs in organoids (*n* = 3 in each group, 2-way ANOVA).

As our attempts to achieve free ASO uptake or ASO transfection using Lipofectamine in kidney organoids were ineffective, we modified the oligonucleotide chemistry from 2′-*O*-Methyl–phosphorothioate (2′-OMe–PS) RNA to phosphorodiamidate morpholino oligomers (PMOs), which are neutral in charge and, owing to their chemical properties, can be more easily taken up by various cell types within the organoids. Organoids were exposed to 5 μM PMO with Endo-Porter (6 μL/mL) to promote endocytic uptake (Figure 7A). To assess the efficacy of PMO penetration within the organoid structure, fluorophore-labeled PMO was used (single transfection for 3 days at day 18). The untreated organoids exhibited no green fluorescence, confirming the absence of PMO (Figure 7B, top right). Conversely, labeled PMO treatment resulted in positive signals from different cell types, mainly in PECs and tubular epithelial cells, and also in podocytes but with a weaker signal (Figure 7B, bottom). Thus, we decided to use longer periods of treatment to improve transfection in podocytes. The timeline is illustrated in Figure 7A, with transfection starting on day 14 and renewed every 3 days until the final analysis on day 38. The experimental groups included isogenic controls treated with scramble-labeled PMO, severe and moderate XLAS models, and their respective PMO-treated counterparts. We also performed splicing outlier analysis using FRASER on bulk RNA-seq data from PMO-treated organoids and did not detect any marked off-target events following PMO treatment.

**Figure 8:**
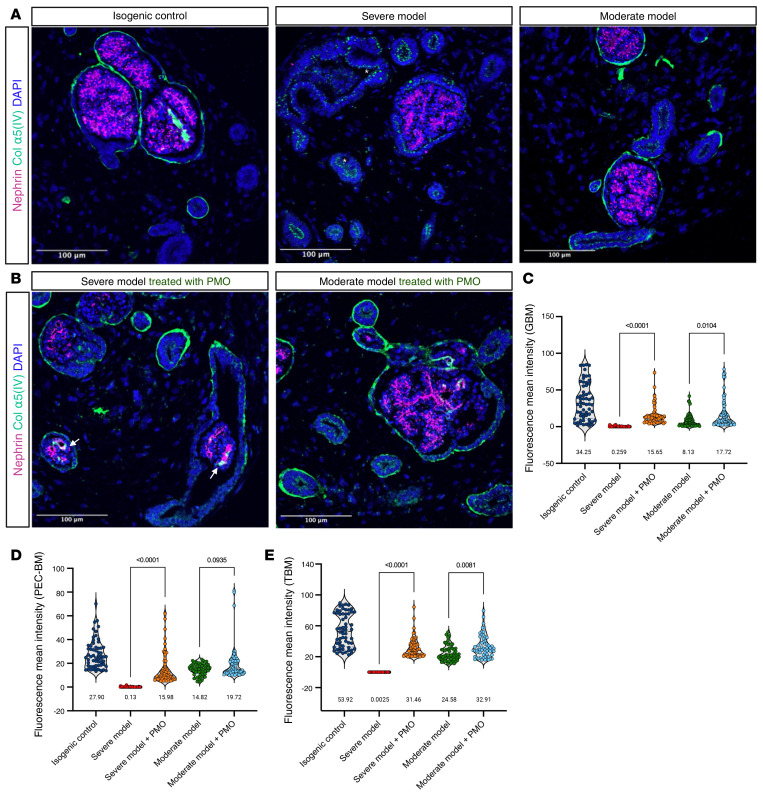
Restoration of BM collagen network in XLAS organoid models following splice modulation therapy. (**A** and **B**) Immunofluorescence staining for collagen α5(IV) and nephrin displaying substantial restoration of collagen α5(IV) production and secretion as a network in the BMs of XLAS organoid models following PMO treatment (the white arrow points to the GBM α5(IV)). In the XLAS severe model before PMO treatment, α5(IV) protein was retained in the apical side of the epithelial cells. Restoration of collagen α5(IV) in the GBM confirmed the penetration of PMO in cells after 3 weeks of treatment. The asterisk refers to the α5(IV) retention inside the cell. Scale bars: 100 μm. (**C**–**E**) Quantification of collagen α5(IV) fluorescence intensity within the GBM (**C**), PEC-BM (**D**), and TBM (**E**) showed significant increases in protein secretion and assembly following PMO treatment in both models (mainly in the severe model) compared with untreated controls. Values below each condition indicate the mean intensity (*n* = 60 objects, ANOVA with Tukey’s multiple-comparison test).

The efficacy of PMO treatment was first evaluated by assessment of the expression levels of WT and mutant *COL4A5* transcripts in whole organoid lysates (Figure 7C). In the severe model, the untreated samples displayed aberrant splicing, while PMO treatment led to a shift toward normal splicing, indicated by dramatically reduced aberrant transcript levels and the emergence of WT mRNA (0% WT junction before treatment, 85.15% after treatment). A splice modulation pattern was observed in the moderate model (58.93% WT junction before treatment, 92.83% after treatment), and PMO treatment significantly decreased the synthesis of mutant *COL4A5* transcripts (Figure 7C, middle panel). Quantitative fragment analysis confirmed the significant increase in the WT/mutant ratio (80.33% in severe model, 86.37% in moderate model) following PMO treatment (Figure 7D). Immunofluorescence staining on organoids proved the efficacy of PMO treatment on the type IV collagen network deposition in different BMs in both severe and moderate XLAS models (Figure 8, A and B). In the untreated severe model, there was no α5(IV) deposition in BMs. PMO treatment significantly restored α5(IV) production, assembly with α3(IV) and α4(IV), and secretion into the GBM (mean intensity = 15.65), TBM (mean intensity = 31.46), and PEC-BM (mean intensity = 15.98) in the severe model. Similarly, in the moderate model, untreated samples exhibited low α5(IV) expression (TBM = 24.58, PEC-BM = 14.82, and GBM = 8.13), which was substantially improved after PMO treatment (mean intensity in TBM = 32.91, PEC-BM = 19.72, and GBM = 17.72). Even though PMO uptake was observed mainly in PECs and tubular epithelial cells, the significant (adjusted *P* value < 0.009) improvement in collagen IV deposition in the GBM of the severe model confirmed successful PMO uptake by podocytes. The quantitative analysis of mean fluorescence intensity (Figure 8, C–E), using our protein quantification method, showed significant increases in collagen α5(IV) deposition, particularly in the severe model and, to a lesser extent, in the moderate model.

Thus, the quantification and analysis of collagen α5(IV) at both the mRNA and the protein level before and after PMO treatment confirmed the effectiveness of using kidney organoids as scalable and robust in vitro models for developing ASO-based therapeutic approaches in XLAS.

## Discussion

Recent advances in integrative genome and transcriptome analyses have substantially improved the diagnosis of genetic diseases, particularly by identifying pathogenic variants that affect splicing. In total, it is estimated that around 20% of the variations identified in monogenic diseases are splicing-related variations (29). In silico scores have a low positive predictive value, and functional studies in valuable models are therefore essential. Moreover, steric-block oligonucleotide therapy has gained attention as an approach to treat various diseases caused by this class of variants (30). We and other groups have previously shown the importance of variants affecting *COL4A5* splicing in XLAS (intronic or exonic) (12, 31–33). In this study, we developed a two-step preclinical ASO assessment framework, using both patient-derived primary cells and kidney organoids featuring patient-specific variations. Then, using a multiomics approach including single-cell RNA-seq and proteomics in combination with confocal imaging–based protein quantification, we deeply characterized the development of the GBM of kidney organoids, WT or carrying the pathogenic *COL4A5* variants identified in two XLAS patients. We showed that prolonged culture of human iPSC–derived (hiPSC-derived) kidney organoids is crucial for ECM maturation, involving both BM components like α3 and 4 chains of type IV collagen and modifying enzymes such as LOXL2. This enzyme is necessary for collagen IV cross-linking in the BMs (34). Using ASOs, we then significantly re-expressed α5(IV) protein in the GBM of kidney organoids, thus opening the door to targeted therapy in these patients. The XLAS organoid development and splice modulation within 2D and 3D models emerged as a pivotal aspect of this study.

Organoids are an invaluable model for evaluating disease pathophysiology and therapeutic options, owing to their genetic similarity to humans and their ECM organization similar to that of kidney tissue (35, 36). Deep characterization of late matured kidney organoids following specific differentiation protocols is missing. Here, we used multiomics (bulk and single-cell RNA-seq and proteomics) and immunofluorescence staining on organoids harvested at early (day 22), mid (day 32), and late (day 38) time points. An integrative analysis of bulk RNA-seq and proteomics data confirmed dynamic changes in ECM- and cell junction–related genes and proteins when comparing early (day 22) with late (day 38) time points (37). Notably, consistent with recent observations (35), *COL4A3*, *COL4A4*, and *COL4A5* expression was significantly enriched in organoids harvested at day 38, confirming the collagen switch during GBM development. Moreover, analysis of single-cell RNA-seq data (from day 22 and day 38) revealed upregulation of *COL4A3* and *COL4A4*, favoring GBM maturation. We identified distinct podocyte populations in the datasets, from early podocyte progenitors to “more” differentiated podocytes. By day 38, progenitor populations had decreased, giving rise to a more mature podocyte profile. Immunostaining analysis of type IV collagens in the GBM at early and late time points confirmed increased mature collagen deposition, which was not investigated before. All these findings revealed the importance of maintaining organoids in culture for a longer period to have more mature GBM, which is necessary for XLAS modeling.

Focused analysis of the podocyte cell population in the single-cell data identified pathways and ontology terms that could explain the cell type–specific pathology of the disease. The downregulation of BM genes such as *COL4A5*, together with loss of adhesion-related genes including *MAGI1*, suggests compromised structural integrity and altered podocyte-matrix interactions (38). Concurrent cytoskeletal remodeling, marked by reduced expression of stabilizing genes such as *TPM1* and *ARHGAP24* and increased expression of the stress-associated genes *HTRA1* and *AIF1*, indicates a shift toward an injury-responsive state (39, 40). The downregulation of PDGF-, VEGF/NRP1-, MAPK-, and Ca²^+^-associated genes in diseased podocytes is consistent with prior evidence that suppression of these pathways impairs podocyte survival and mechanosensing (41), while the upregulation of mitochondrial respiratory and proteostasis-related genes reflects activation of stress-adaptive programs, such as mitochondrial biogenesis and unfolded protein response, previously shown to be engaged in podocytes under mechanical or metabolic stress (42–44).

Patient-derived cells most often allow analysis of transcription of the patient’s pathogenic variants. This is only possible if the gene is expressed in accessible cells, which is the case for the *COL4A5* gene in fibroblasts but is not the case for the *COL4A3* and *COL4A4* genes not expressed by fibroblasts, since only the α5α6α5(IV) chains are expressed at the dermo-hypodermal junction. Thus, the kidney organoid model adds an important dimension by measuring the impact on the protein encoded by this gene. In particular, in AS, the ability not only to study the precise localization of α5(IV) chain on a cellular level but to quantify its level offers a perfect readout.

After demonstrating the capability of prolonged culture of kidney organoids to recapitulate key features of AS at the BM level, we sought to develop a framework for transfecting ASOs in kidney organoids in order to modulate *COL4A5* mRNA splicing. Different groups have proved the applicability of ASOs in different types of organoid models. A study has used ASOs (1 μM) for splice modulation in hiPSC-derived cortical and subpallial organoids and observed effects on calcium channel function and gene expression (45). Another study generated patient-derived organoid of pancreatic ductal adenocarcinoma and tested free uptake and transfection-based ASO treatment and concluded that transfection-based delivery is more effective than free uptake (46). Very recently, a study evaluated ASO-mediated exon skipping in kidney organoids bearing *COL4A5* pathogenic variants. ASO treatment improved α5(IV) chain deposition in vitro, but after transplantation of organoids at different induction stages, efficient restoration of collagen expression was not achieved, despite evidence of vascularization (47). In our study, transfection of ASOs without carrier and with carrier was not efficient in penetrating the organoids. Therefore, we shifted toward the PMOs because of their neutral charge.

Recent research on PMO-based splice modulation in organoid models offers valuable insight into their therapeutic potential. Hall et al. generated colorectal cancer organoids from mouse models and patient samples and used PMOs to inhibit RNA splicing targeting KRAS isoforms (*KRAS4B*). This approach notably decreased tumor growth by altering splicing in cancer stem cell pathways (48). Parfitt et al. used an optic cup organoid model harboring the common *CEP290* deep-intronic variant and treated these organoids with PMOs targeting this variant (49). They treated their organoids every 3 days for 4 weeks beginning at week 13 of organoid development. This treatment led to a significant increase in the WT *CEP290* transcript (70%). Other researchers generated retinal organoids from hiPSCs to induce exon skipping in the *PROM1* gene. PMOs were applied to these organoids at a concentration of 10 μM, with treatment conducted weekly over a 4-week period, achieving around 57% exon skipping (50). This research showed the possibility of using RNA- or morpholino-based oligonucleotides in organoid models. Consistent with these successful studies, our study demonstrated that PMOs are highly efficient in restoring correct *COL4A5* splicing in both severe and moderate XLAS organoids.

In 2020, Yamamura et al. demonstrated the applicability of ASOs to induce in-frame skipping of an exon harboring a pathogenic stop variation in a mouse model of XLAS (15). Despite successful restoration of collagen IV networks in the GBM and TBM, this treatment strategy aimed only to reduce a severe disease phenotype to a less severe one, since the full protein correction could not be achieved. However, splice-switching therapy offers the potential to completely reverse the phenotype by producing an intact, functional protein, when aimed at intron retention suppression. Our approach to this aim involved analyzing the splice site recognition sites and masking a few regions to find the best target with the highest WT/mutant ratio after treatment while minimizing its off-target activity. The effectiveness of our approach was proven through experiments conducted on primary cells, then on organoids. Our results confirmed the efficacy of ASOs in restoring normal *COL4A5* splicing in multiple patient-derived models without showing notable off-target effect on mRNA level. Furthermore, our prolonged cultured kidney organoid model and the framework of analyses, including rescue by ASOs described herein, can ultimately be generalized to the α3(IV) and α4(IV) chains expressed by kidney organoids, but also to other key proteins of the podocyte or other kidney cells with specific membrane localization and in which we have identified intronic variations (51, 52).

While kidney organoids are invaluable models for studying disease mechanisms and therapeutic strategies, they still face some limitations. Firstly, a key limitation is the absence of vascularization of kidney organoids, which remains a challenge in fully mimicking in vivo kidney properties, especially GBM integrity. Vascularization is crucial for enhancing podocyte and GFB maturation and integrity, and can also improve the efficacy of ASO delivery to targeted podocytes. Encouragingly, recent advancements in organoid culture devices, such as flow systems, offer promising opportunities to overcome this limitation (53–55). Incorporating vascularization strategies could further refine our model, enabling a more accurate disease representation and therapeutic evaluation. Secondly, unlike animal models, kidney organoids cannot recapitulate proteinuria and are limited by certain physiological readouts. However, it has been proven that hematuria and proteinuria are not the only readout in patients and animal models of AS. Indeed, the correlation between the amount of type IV collagen chain in the ECM and disease severity has been proven in AS, in particular in a recent paper in biopsies from patients with AS (56). The correlation between expression levels of α5(IV) and the associated clinical phenotype could in the future make it possible to extrapolate the results obtained in organoids, particularly for assessing the efficacy of therapies, to the results expected in an animal model, or even in patients. This could avoid the need for systematic use of an animal model.

In this study we succeeded in generating a robust framework for modeling XLAS and evaluating ASO-based therapeutic interventions. Our findings highlight the utility of kidney organoids in bridging the gap between basic research and clinical application. As the field of organoid technology continues to advance, we anticipate that these models will play an increasingly pivotal role in the development of precision therapies for genetic disease.

## Methods

### Sex as a biological variable.

Sex as a biological variable was considered in the study design. However, because XLAS is caused by pathogenic variants in the *COL4A5* gene located on the X chromosome, all experiments were performed using iPSC lines derived from male donors. This approach avoids variability related to X chromosome inactivation and mosaic expression of *COL4A5* in female cells, and allows a direct assessment of disease mechanisms associated with hemizygous pathogenic variants.

### Primary cell and hiPSC culture and maintenance.

Patient-derived fibroblasts were cultured at 37°C with 5% CO_2_ in RPMI 1640 (Thermo Fisher Scientific) with 10% FBS and 1% penicillin/streptomycin 100 U/mL. In some cases, to inhibit nonsense-mediated mRNA decay, the cells were treated with 30 μM emetine for 16 hours and collected after washing with 1× PBS.

The hiPSCs were cultivated in mTeSR Plus (StemCell Technologies) on plasticware precoated with Vitronectin XF (StemCell Technologies) or Matrigel hESC-Qualified Matrix (Corning). The passaging of the cells was made once a week manually under a stereomicroscope (cutting/scraping) or with RelesR reagent (StemCell Technologies). We used Cryostor CS10 to freeze them (StemCell Technologies). The hiPSC line IMAGINE004 was generated by the IPS facility of the Imagine Institute from commercial male PBMCs (StemCell Technologies) using Sendai virus procedure (56).

### CRISPR/Cas9–mediated knockin in the COL4A5 gene.

A V5 tag was inserted at the beginning of exon 2 of *COL4A5* at position c.384 in the IMAGINE004 iPS cell line, and subsequently, patient mutations c.277–560T>G and c.609–879A>G were inserted into the resulting iPS cell line via CRISPR/Cas9 genome editing strategy (Supplemental Figure 6). We chose the closest CRISPR RNAs (crRNAs) to the variation site from the CRISPOR website (https://crispor.gi.ucsc.edu/), and we designed 60-nucleotide single-stranded oligodeoxynucleotide (ssODN) with phosphorothioate modifications for each insertion (Supplemental Table 8). In brief, 300 pmol of gRNA (Alt-R CRISPR/Cas9 crRNA XT and Alt-R CRISPR/Cas9 tracrRNA ATTO 550) and 150 pmol of Cas9 (Alt-R S.p. HiFi Cas9 Nuclease V3) were mixed to form ribonucleoprotein (RNP) complexes (Integrated DNA Technologies). Then, 600 pmol of ssODN was added to the RNP mix. Before nucleofection, hiPSCs were pretreated with the apoptosis inhibitor Y-27632 (10 μM) for 2 hours and further dissociated with TrypLE Select (Thermo Fisher Scientific). Cells were nucleofected with the RNP complexes using the P3 Primary Cell 4D-Nucleofector X Kit-S and 4D-Nucleofector X-Unit (Lonza), using the program CA-137. The cells were then transferred to Matrigel-precoated 24-well plates and cultured in mTeSR Plus medium with 10 μM Y-27632. After 2 days, 10% of the transfected cells were sorted at 1 cell per well in 96-well plates precoated with CellAdhere Laminin-521 (StemCell Technologies). Cells were incubated in mTeSR Plus medium supplemented with CloneR2 (StemCell Technologies) for 3 days. We used Quick-extract DNA Extraction solution (Biosearch Technologies) and Sanger sequencing to identify clones of interest. The absence of genetic rearrangements subsequent to CRISPR/Cas9 genome editing technology was verified using an SNP array (Imagine Institute, iPSC platform).

### Generation of kidney organoids from hiPSCs.

We optimized a previously published protocol (16) to generate kidney organoids using hiPS cell lines. Human iPSCs (4,000 to 8,000 cells/cm^2^) were dissociated with TrypLE Select, and plated onto Matrigel-coated plates (Corning Matrigel Basement Membrane Matrix Growth Factor Reduced). We added Matrigel on the hiPSCs 1 day after their seeding (0.125 mg of protein/mL in mTeSR Plus) to induce the formation of iPSC spheroids during 3 days without any media change. Four days after seeding, the cells reached 40%–60% confluence, and differentiation was initiated (day 0) using Basal Differentiation Medium (BDM) (Advanced RPMI 1640), 1× L-GlutaMAX, and 0.5% penicillin/streptomycin (Thermo Fisher Scientific). Then, we used BDM supplemented with Noggin (PeproTech) and Chiron99021 (Axon Medchem) for 4 days, 4 μM on the first day to reduce cell death and then 8 μM; and then BDM with Activin A 10 ng/mL (PeproTech) for the next 3 days. From day 7 to day 14, FGF-9 was added at 10 ng/mL (R&D Systems), with media changes every 2 days. By day 9, we added 3 μM Chiron99021 for 2 hours or 4 hours depending on the number of renal vesicles observed. For 3D culture, cells were dissociated by day 10 with Accutase (StemCell Technologies) and plated at 25,000 cells per well onto “ultra-low-adherent” 96-well plates with U-shaped bottoms (Corning) in the BDM. Differentiation continued in the same way from day 10 to day 42, with media change every 2 days.

### Histology and immunostaining.

Organoids were immersed in 4% paraformaldehyde (PFA) at 4°C for 20 minutes and washed twice with 1× PBS without calcium and magnesium, then stored in PBS at 4°C. To prepare formalin-fixed, paraffin-embedded (FFPE) sections, the PFA-fixed organoids were embedded in 2% agarose gel, enabling them to be embedded in paraffin, and cut into 4 μm sections. For immunofluorescence microscopy, the FFPE tissue samples were subjected to a serial deparaffinization process, involving two 10-minute deparaffinization steps, two 5-minute steps in 100% ethanol, 5 minutes in 90% ethanol, and an incubation in 80% ethanol, followed by washing in Milli-Q water (Milli-Q Direct Water Purification System, MilliporeSigma). FFPE samples underwent heat-induced antigen retrieval by immersion in 10 mM sodium citrate buffer (pH 6.0) in a rice cooker for 20 minutes. Then, they were treated with a 0.1 M glycine/6 M urea solution for 30 minutes at room temperature. The deparaffinized sections were subjected to nonspecific binding site blocking with 1× casein in PBS and treated with primary and secondary antibody solutions (Supplemental Table 9). The slides were mounted using Mowiol (in-house) and analyzed using a Zeiss Z1 spinning disk confocal microscope at the Imagine Institute imaging platform. For protein quantification in organoids, glomeruli and tubule annotations were made and classified using QuPath (v0.5.1) (57) and SAM API (v0.6.1) (58) and manually corrected. These annotations were then exported as regions of interest (ROIs). Then α5(IV)-positive signal in each image was identified using Shallow Learning pixel classification with Ilastik (v1.4.0) (59). We finally measured α5(IV) mean intensity in glomerulus border and interior, using the ROIs and the Ilastik pixel classification models generated before, with a Fiji (v2.14.0) (60) macro. Refer to the GitHub page for more information (https://github.com/hassansaei/BMQuant).

### RNA extraction, cDNA synthesis, and RT-qPCR.

Total RNA was extracted from both organoids and primary cells using the RNeasy Mini Kit followed by DNase treatment. Single-strand complementary DNA (cDNA) was synthesized from 500 ng or 1,000 ng total RNAs using SuperScript II Reverse Transcriptase (Thermo Fisher Scientific). Quantitative reverse transcription PCR (RT-qPCR) analysis of target genes was performed using iTaq Universal SYBR Green Supermix (Bio-Rad) on a Bio-Rad CFX384 instrument. The relative gene expression was calculated using the 2^–ΔΔCt^ method. The primers used for RT-PCR and RT-qPCR analysis are provided in Supplemental Table 10.

### Transcript analysis using FAM-labeled primers.

For conventional PCRs involving gel screening and fragment analysis, a pair of variant-specific primers with forward primers labeled with 6-FAM were used. The optimal number of 30 PCR cycles for patient fibroblasts and organoids was established using qPCR. Fragment analysis was performed with 1 μL of PCR product using ABI 3500xL fragment analyzer (Applied Biosystems). The area under the curve value was calculated using Peak Scanner software (Thermo Fisher Scientific) to determine the quantity of *COL4A5* WT and mutant transcripts.

### Targeted RNA sequencing.

To prepare libraries for targeted RNA-seq, first-strand cDNA was synthesized from total RNA using the SuperScript VILO kit (Thermo Fisher Scientific), and the second-strand cDNA synthesis kit (Thermo Fisher Scientific) was used to produce the second strand. The double-stranded cDNAs were purified using AMPure XP Reagent (Beckman Coulter), and 50 ng of cDNA was used to prepare the next-generation sequencing targeted RNA-seq libraries using a TWIST technology. The capturing probes were synthesized from a long-range PCR on the *COL4A5* full coding sequence in the plasmid pNLF1-C_hCOL4A5-Nluc (198113, Addgene) (61). Fragmentation of cDNA was carried out using the Twist Library Preparation EF kit and Twist Universal Adapter System kit, and biotinylation of the probes was performed using an in-house protocol. Paired-end sequencing (150 bp + 150 bp) was performed using a MiSeq sequencer (Illumina). The FASTQ files were aligned to the GRCh37 assembly of the human genome using Hisat2 software (https://daehwankimlab.github.io/hisat2/), and the visualization of the junctional reads was performed using the Sashimi plot view feature of the IGV software (Broad Institute).

### Bulk RNA sequencing and data analysis.

Total RNA was extracted from the pool of 9 organoids per condition (3 time points: days 22, 32, 42; and ASO-treated and untreated control and diseased organoids with 3 replicates in each). The quantity and quality of the extracted RNAs were assessed using the Lunatic nucleic acid quantification system (Unchained Labs) and an Agilent Technologies 5200 Fragment Analyzer. The library was constructed with approximately 200 ng of total RNA using the Universal Plus mRNA-seq Kit (Tecan). The 24 libraries were sequenced on the NovaSeq 6000 platform (Illumina) using the S1 flow cell at the genomics platform at the Imagine Institute. The FASTQ files were aligned to the Ensembl GRCh38 assembly of the human reference genome using the Illumina DRAGEN Bio-IT Platform (v4.0), and the gene count matrix was generated using the featureCounts software (62) in R. The matrix of counts was analyzed using 3 independent statistical methods: edgeR (63) (v3.26.8), DESeq2 (64) (v1.24), and limma-Voom (65) (v3.40.6). The relevant genes were identified from each comparison group using an absolute logarithm fold change (logFC) greater than 1.2 and the adjusted *P* value (*P* < 0.05). Gene enrichment or over-representation analysis was conducted in R using the enrichment function of the ClusterProfiler (66) package. Aberrant splicing events in disease models with and without ASO treatment were analyzed by FRASER (67). Heatmaps and regular and bar plots were generated in R using pheatmap, tidyverse, and ggplot2 packages, respectively.

### Sample enrichment for proteomic profiling.

Kidney organoids harvested at days 22, 32, and 38 of differentiation (*n* = 16 organoids pooled) were subjected to cellular soluble protein and matrix protein enrichment, as previously described (4). Samples were washed 3 times with 1× PBS, then homogenized in Tris–lysis buffer (10 mM Tris [pH 8], 150 mM sodium chloride, 1% Triton X-100, 25 mM EDTA in LC/MS–grade water, and an EDTA-free Roche complete protease inhibitor cocktail). The organoids were incubated in this solution for 1 hour at 4°C with constant rotation and centrifuged at 14,000*g* for 10 minutes at 4°C. The supernatant was then collected and labeled as fraction 1 (enriched with soluble cellular proteins). The pellet from the first step was resuspended in an alkaline detergent buffer (0.5% Triton X-100, 0.1 M PBS, and 20 mM ammonium hydroxide). Fraction 2, which contained the cell surface and transmembrane proteins, was collected following the same process as fraction 1 (incubation and centrifugation to obtain the supernatant). The remaining pellet was resuspended in 50 μL of TEAB/SDS lysis buffer (100 mM TEAB [pH 8.5], 10% SDS), and the samples were subjected to sonication using Covaris AFA glass tubes in the Covaris LE220+ system to obtain fraction 3 (ECM fraction). The sonication parameters for kidney organoids were set as follows: 40 seconds per sample, peak power of 500 W, duty factor of 20%, 50 cycles per burst, and average power of 100 W. Fractions 1 and 3 were analyzed separately. To reduce the samples, 5 μL of a 100 mM dithiothreitol solution was added to 100 μL of the soluble fraction and heated 10 minutes at 60°C. After cooling, 15 μL of 100 μL iodoacetamide solution was added to the fractions for 30 minutes at 4°C to alkylate the proteins. Sample preparation for label-free mass spectrometry–based proteomics involved overnight digestion of samples with Trypsin Gold (Promega) in S-Trap Spin Columns (Protifi, C002-MICRO-0010PK), followed by purification using OLIGO R3 Reverse beads (Thermo Scientific) in acetonitrile. The resulting peptides were dried and subjected to mass spectrometry data acquisition using a Thermo Fisher Scientific Q Exactive HF hybrid quadrupole-Orbitrap mass spectrometer with a Dionex Corp. UltiMate 3000 Rapid Separation LC nanosystem at Manchester University.

### Bioinformatic analysis of the proteomics data.

We used MaxQuant (MQ) software (v2.4.9) on the biocluster server at the Imagine Institute, which was operating under dotnet version 3.0 (Microsoft), to analyze the raw proteomics data. Separate MQ parameter files (mqpar.xml) were generated for the cellular fraction and ECM data, with carbamidomethylation of cysteine as a fixed modification and oxidation of methionine, proline, and lysine and N-terminal acetylation as dynamic modifications. These modifications were added to improve the collagen peptide identification. The MS data were searched against UniProt and TrEMBL databases for humans (txid, 9606). MQ produces a separate peptide file for each analysis, including cellular fraction and ECM fraction. Perseus software (https://maxquant.net/perseus/) was used to merge cellular with ECM peptides, and data normalization and statistical analysis were performed. A multidimensional scaling plot was generated to perform a quality check. Differentially expressed proteins were filtered based on the adjusted *P* value (< 0.05), and the differentially regulated proteins were visualized in R using ggplot2 function.

### Organoid dissociation and single-cell RNA sequencing.

Two batches of samples were collected. In batch 1, 16 organoids were harvested at day 22 from isogenic control, severe XLAS, and moderate XLAS lines (3 libraries). Two additional isogenic controls were collected — one at day 22 and one after prolonged culture to day 38 — yielding 5 libraries in total. Organoids were washed in PBS and dissociated by repeated 10-minute incubations in TrypLE at 37°C with gentle mixing; supernatants were quenched in PBS/10% FBS, and mechanical pipetting was introduced from the third round. Pooled dissociated cells were filtered through a 40 μm strainer, pelleted (300*g*, 3 minutes), resuspended in PBS with 1% BSA and 1 mM EDTA, and kept on ice, with viability ranging from 70% to 85%. Libraries were generated using the Chromium Next GEM Single Cell 3′ and Chip G kits (10x Genomics) at the Institut du Cerveau (Paris, France) genomics platform, assessed using the High Sensitivity D5000 ScreenTape (Agilent Technologies), and sequenced on NovaSeq X with the X Plus 10B Reagent Kit (both from Illumina) (100 cycles).

### Single-cell RNA sequencing data analysis.

The sequencing data were preprocessed using the CellRanger pipeline (10x Genomics, Cellranger count v7) with default parameters, aligned to GRCh38, and the resulting matrix files were analyzed using the Seurat (v5.1.0) package in R (v4.3.3) software. Outliers were detected and filtered using a function that defined the median absolute deviation (MAD) with the number of MAD (nMAD) set to 5 for nCount_RNA and nFeature_RNA (both log1P) and 3 for the percentage of mitochondria. The SoupX (68) algorithm was used to remove ambient RNAs, and the DoubletFinder (69) algorithm was used to remove potential doublets. The Seurat object’s assay version was set to “v3” to use older reduction and data integration methods (CCA and RPCA). Integration features (nfeatures = 3,500) were selected, followed by the application of PrepSCTIntegration. Next, the integration anchors were identified using CCA reduction method, and the datasets were integrated using the IntegrateData function. Dimension reduction was performed using RunPCA, and clusters were determined using resolutions, from 0.5 to 1.6. RunUMAP functions were applied to the integrated datasets, and the best resolution was selected based on the number of unique informative clusters. To compare organoids at 2 time points (day 22 and day 38), we applied the CCA method and uniform manifold approximation and projection (UMAP) with a resolution of 1.2. The scCustomize library was used to select a broader range of palettes for data visualization. The final cell type annotations were added using knowledge-based methods, which involved extracting differentially regulated markers (using FindMarker) for each cluster and incorporating literature-based annotations (70, 71). To compare mutants with the isogenic control, we used CCA integration with UMAP visualization of clusters identified with a resolution of 1.6. The DESeq2 (64) method was applied to a data frame obtained by subsetting of the podocyte clusters and pooling of the reads in each sample using the AggregateExpression function. We used the DotPlot function in the Seurat package to produce dot plots. Heatmaps were generated using the Pheatmap function. We used UCell to perform rank-based gene signature enrichment analysis (27).

### Designing antisense oligonucleotides for splicing modulation.

Antisense oligonucleotides (ASOs) with 2′-*O*-methylation (2′-OMe) RNA chemistry and a full phosphorothioate backbone were designed and synthesized by Eurogentec and purified by reversed-phase HPLC. The ASO binding location was chosen, and the final sequence was determined by the probable off-target score obtained from the NCBI BLAST engine. The ASO that binds to the splice acceptor site is called ASO-A; to the donor site, ASO-D; and to the site with a variation, ASO-M. The ASOs that bind to both the variation sites and either the acceptor or the donor sites are named ASO-MA or ASO-MD, respectively. When two ASOs were used in combination, the (+) was added between ASO IDs. To treat organoids, phosphorodiamidate morpholino oligomers (PMOs) and Endo-Porter peptides with polyethylene glycol conjugates were ordered from Gene Tools, maintaining the same sequence as the ASO tested in patient-derived primary cells. Scramble PMO with 3′-carboxyfluorescein was used to monitor the uptake by various cell types in the organoid.

### Transfection of patient-derived primary cells with ASO.

Patient-derived fibroblasts were cultivated and seeded in the 6-well plates for transfection. The RPMI 1640 medium was removed from the plate and washed once with 1× PBS and Opti-MEM (Gibco). The transfection solution (500 μL Opti-MEM, 7 μL Lipofectamine (Invitrogen), and 1.5 μL ASO [100 μM stock concentration]), consisting of effective ASOs or scramble ASOs, was added to the medium dropwise and incubated for 6 hours before the medium was changed back to high-serum regular medium.

### Morpholino treatment in kidney organoids.

PMO transfection solution was prepared by mixing of 5 μM of each PMO with 1 mL of BDM and 6 μL of Endo-Porter peptide to facilitate efficient cellular uptake. Transfection mix was added to each organoid in a 96-well ultra-low-attachment plate (100 μL per well). The treatment protocol lasted for 3 weeks, with medium changes and transfections every 3 days continuing until day 38. The study included control groups, such as untreated organoids and organoids treated with labeled PMO, to evaluate transfection efficiency.

### Transmission electron microscopy.

Organoids were fixed in 2.5% glutaraldehyde solution for 2 hours, washed with 1× PBS three times, and stored in 4% PFA in PBS before processing in the Electron Microscopy Platform at Manchester University. Organoids were washed with HEPES buffer (0.1 M, pH 7.2) several times, then stained with reduced osmium (1.5% potassium ferrocyanide and 1% osmium tetroxide in 0.1 M cacodylate buffer, pH 7.2) for 1 hour and with 1% uranyl acetate in water overnight. They were dehydrated in ethanol or acetone series, embedded into TAAB Low Viscosity Resin, and polymerized at 6°C for 24 hours. The 70 nm sections were cut with a Leica UC7 ultramicrotome and placed on a 1-by-2-mm copper slot grid coated with formvar/carbon film. Images were taken with a Thermo Fisher Scientific Talos L120C electron microscope at 120 kV acceleration voltage using a Ceta camera.

### Statistics.

Statistical analysis for transcript quantification using fragment analysis, RT-qPCR, and macro-enabled protein quantity assessment was conducted using GraphPad Prism (v10.3.0). Multigroup comparisons were analyzed using 1- or 2-way ANOVA with Tukey’s post hoc correction for multiple comparisons. Differentially expressed genes (DEGs) were identified based on an adjusted *P* value threshold using the Benjamini-Hochberg method for false discovery rate (FDR) correction. Principal component analysis (PCA), multidimensional scaling, and unsupervised hierarchical clustering were performed using RStudio, and plots were generated using the ggplot2 package. For all statistical analyses, significance was defined at an adjusted *P* value threshold of <0.05, and details of specific tests (e.g., fold-change cutoffs, threshold settings) are reported in the relevant sections of Results. Data are presented as mean ± SEM in all panels in which error bars are shown.

### Study approval.

All patients gave written informed consent for collection of biomaterials. The research was performed in accordance with the Declaration of Helsinki on human experimentation of the World Medical Association, and it was conducted with the approval of the Comité de Protection des Personnes pour la recherche biomédicale, Île de France.

### Data availability.

The bulk and single-cell RNA-seq data were deposited to the NCBI’s Gene Expression Omnibus database with the dataset identifiers GSE281080 for bulk and GSE281081 for single-cell RNA-seq. The mass spectrometry proteomics data were deposited to the ProteomeXchange Consortium via the PRIDE partner repository (72) with the dataset identifier PXD057360. The Fiji macro for BM protein quantification is provided in the GitHub page (https://github.com/hassansaei/BMQuant). Scripts and workflows used in this study for the analysis of single-cell and bulk RNA-seq and proteomics datasets are available at https://github.com/hassansaei/Saei_JCI_Insight_2025 Additional supplemental datasets and figures were deposited in Zenodo and can be accessed at https://zenodo.org/records/16585561 Values for all data points in graphs are reported in the Supporting Data Values file.

## Author contributions

HS led conceptualization, data curation, methodology, formal and bioinformatic analyses (single-cell, bulk RNA-seq, proteomics), imaging, cell culture and differentiation, visualization, and manuscript writing and editing. BE performed iPSC genome editing and organoid differentiation. NG developed the Fiji macro. ME contributed to stem cell maintenance, organoid work, and molecular analyses. JH assisted with cell culture. OG contributed to writing and review. C Arrondel performed primary cell culture. VM conducted targeted RNA-seq. PT performed proteomics sample enrichment. RL funded proteomics. C Antignac, GM, and GD contributed to conceptualization, funding, methodology, review, and the original draft.

## Funding support

- ORKiD healthcare network (2021, 2023).
- Agence Nationale de la Recherche “Investissements d’avenir” program (ANR-10-IAHU-01).
- HS is a fellow of the Pasteur Paris University–Imagine International Doctoral Program.

## Supplementary Material

Supplemental data

Supplemental table 1

Supplemental table 2

Supplemental table 3

Supplemental table 4

Supplemental table 5

Supplemental table 6

Supplemental table 7

Supporting data values

## Figures and Tables

**Table 1:**
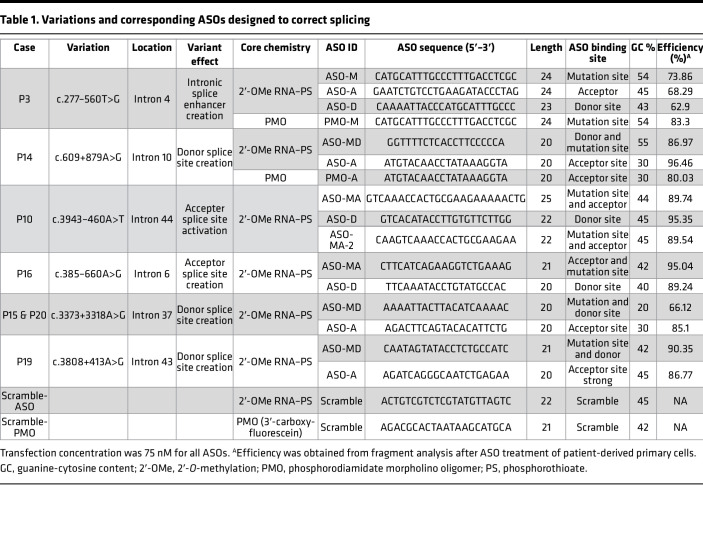
Variations and corresponding ASOs designed to correct splicing

## Version 1 12/18/2025

In-Press Preview

## Version 2 02/09/2026

Electronic publication
