# Supplementary material for: Splice modulation of COL4A5 reinstates collagen IV assembly in an organoid model of Alport syndrome
Source: JCI Insight. 2025 Dec 18;11(3):e194759. doi: 10.1172/jci.insight.194759 (PMC12892906; doi:10.1172/jci.insight.194759)
Supplement: Supplemental data [file jciinsight-11-194759-s140.pdf]

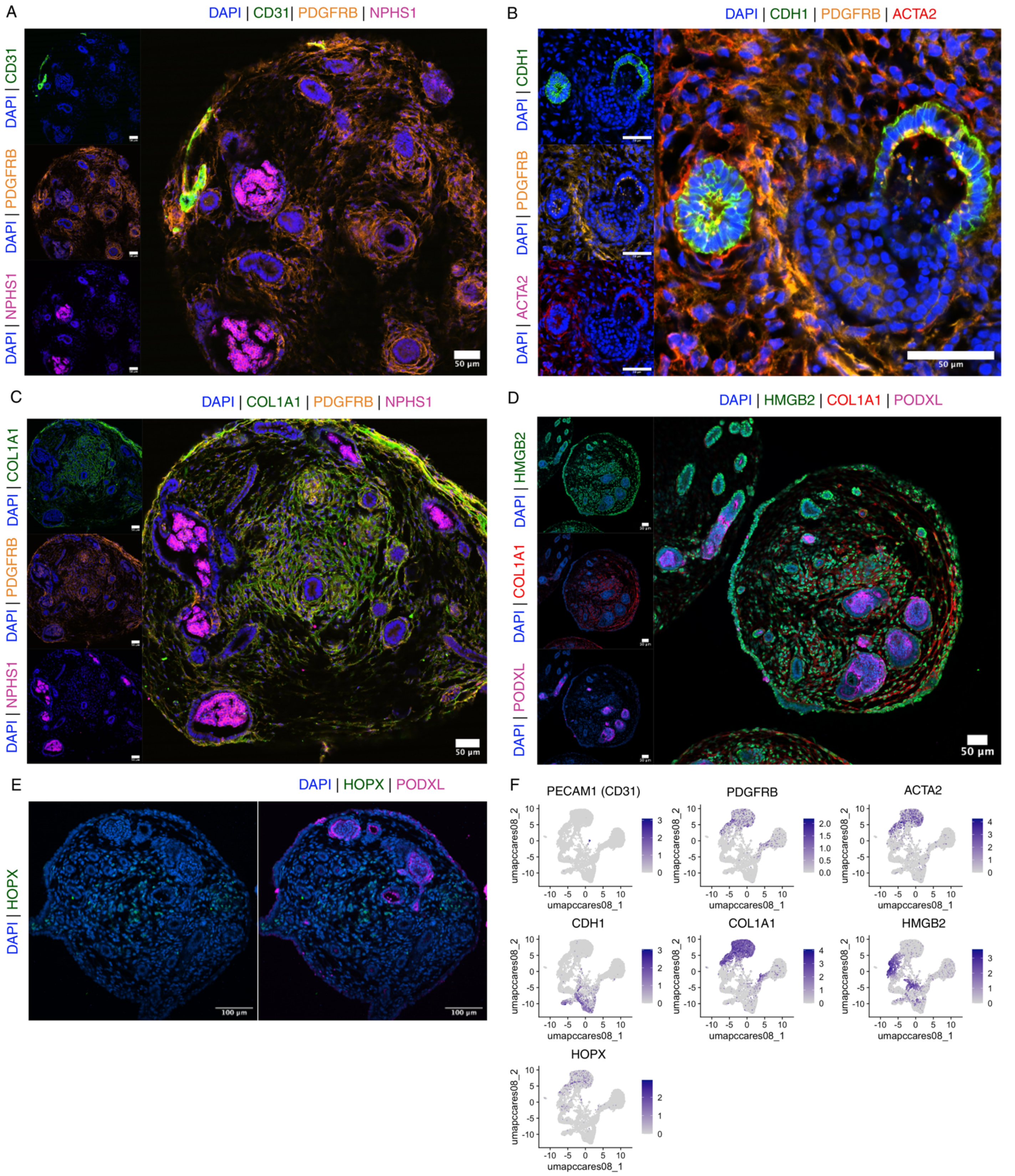

Supplemental Figure 1. Immunofluorescence staining of vascular endothelial cells, fibroblasts, myofibroblasts, proliferative cells and mesangial progenitors in kidney organoids at day 38 of culture. (A) Organoids (OCT cryosections, 20 μm) stained for endothelial cells (PECAM1/CD31$^+$, green) and fibroblasts (PDGFRB, yellow) and podocytes (NPHS1, magenta) (B) Organoids (OCT cryosections, 20 μm) stained for distal tubule epithelium (CDH1, green), fibroblasts (PDGFRB, yellow), and myofibroblasts (ACTA2, red). (C) Organoids (OCT cryosections, 20 μm) stained for stromal matrix (COL1A1, green), fibroblasts (PDGFRB, yellow) and podocytes (NPHS1, magenta) (D) Organoids stained for proliferative cells (HMGB2, green), stromal matrix (COL1A1, red), and podocytes (PODXL, magenta). (E) Mesangial progenitor cells immunostained for HOPX. (F) FeaturePlots highlighting mRNA expression of markers used in the corresponding immunostaining. Sections in part D and E are 4 μm parafilm-embedded sections. Scale bars as indicated on each image.

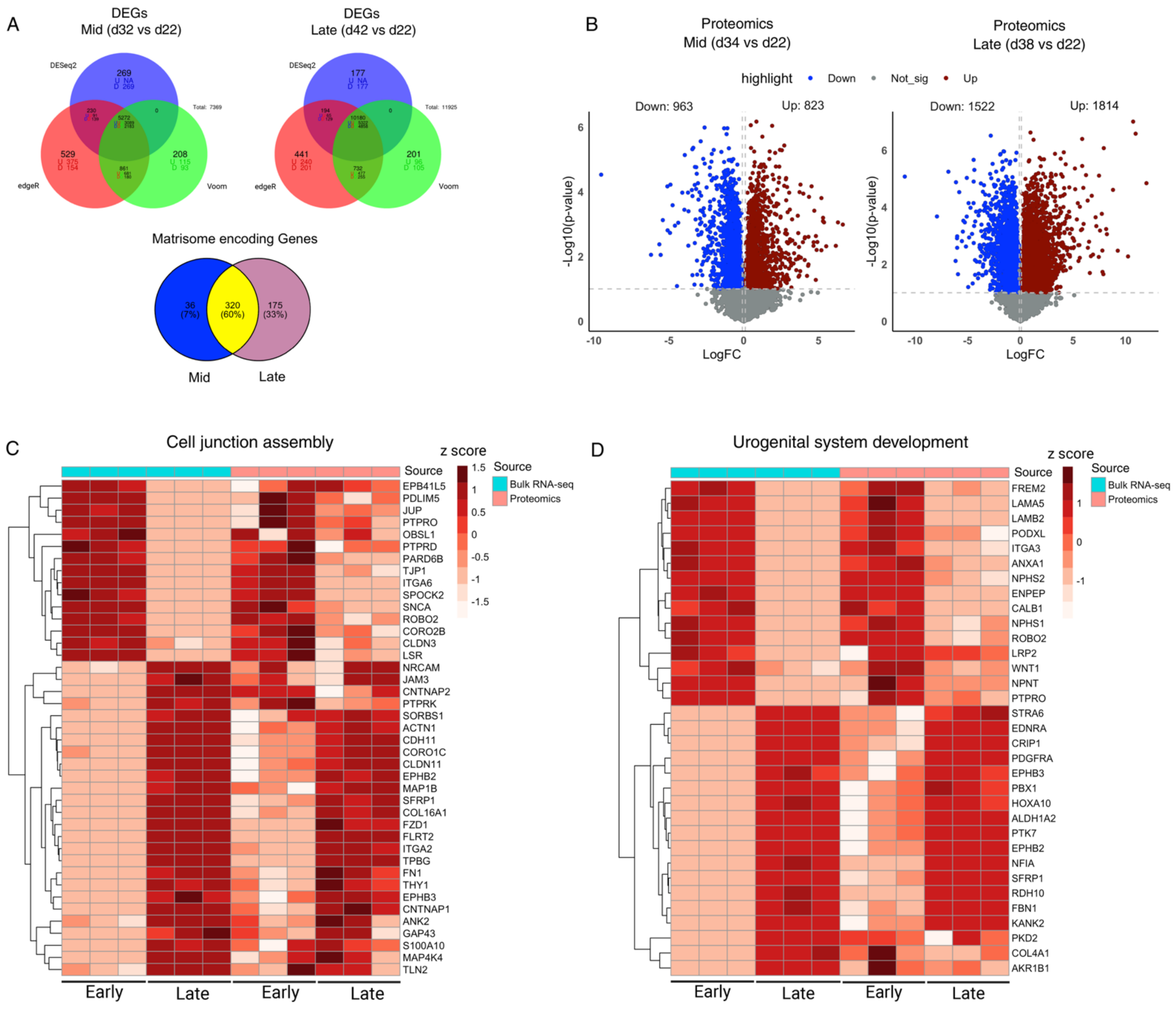

Supplemental Figure 2. Integrative analysis of bulk RNA-seq and global proteomics data. (A) Venn diagrams of DEGs obtained from three different statistical methods (DESeq2, edgeR, and limma-voom, fold change:1.2) at day 42 vs day 22 and day 32 vs day 22. In the d32 vs d22 group, 5,272 DEGs were identified, with 3,089 upregulated and 2,183 downregulated genes. In the d42 vs d22 group, 10,180 DEGs were found, with 5,322 upregulated and 4,858 downregulated genes. We used the gene list from the NABA matrisome library (1,025 matrix protein-encoding genes) to identify differentially regulated ECM-encoding genes in each group, with an overlap in matrisome-encoding genes. A total of 320 shared ECM-encoding genes were identified between d32 vs d22 and d42 vs d22. Additionally, 175 unique genes were enriched at day 42, indicating the evolution of gene expression beyond day 32. (B) We merged protein groups identified from independent soluble fraction and ECM fraction proteomics analyses and identified 1786 differentially regulated proteins (d34 vs d22), and 3336 differentially regulated proteins comparing d38 vs d22. Panels (C) and (D) present heatmaps of genes and proteins enriched in GO terms related to cell junction assembly and urogenital system development, respectively. These terms were enriched following focused analysis of shared differentially expressed genes and proteins from d38 vs d22 comparison.

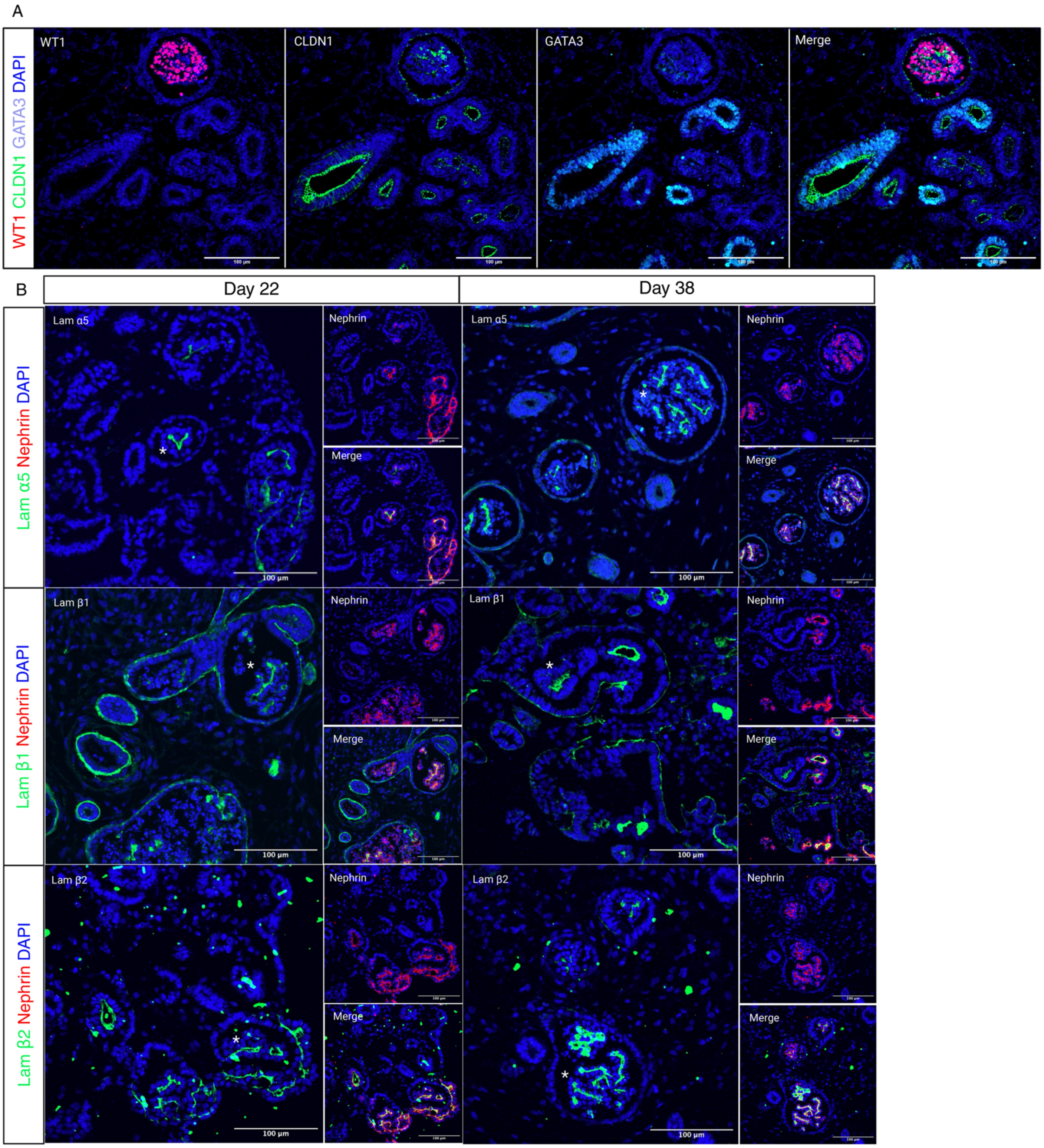

Supplemental Figure 3. Immunofluorescence staining of kidney organoids highlighting the parietal epithelial cells and the localization of different laminins in the BMs. (A) Spatial distribution of WT1 (in red), CLDN1 (in green), and GATA3 (in cyan) in organoid (day 38). As expected, WT1 is mainly localized to the nucleus of the podocytes, while claudin-1 is present in the parietal epithelial-like cells (PEC-like) and distal tubules ($GATA3^{+}$). These cells express collagen type IV network and laminin proteins. Panels in (B) compared laminin α5, β1, and β2 localization and abundance between days 22 and 38 of organoid culture. This panel confirms the presence of laminin mature network in organoids early in the culture.

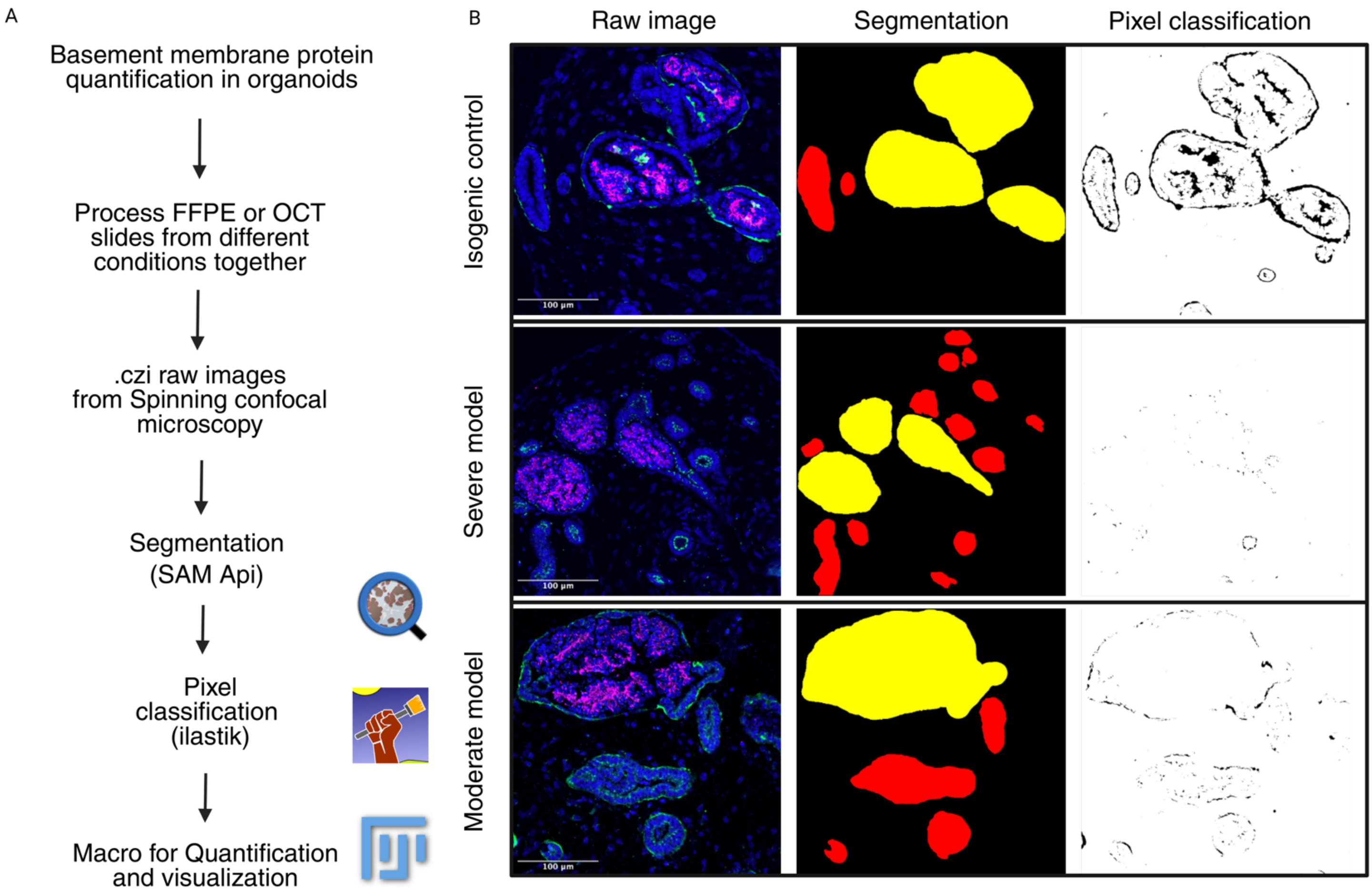

Supplemental Figure 4. Development of a Fiji macro for quantification of basement membrane protein expression in kidney organoids. (A) Basement membrane protein quantification workflow. Glomeruli and tubules are first segmented in QuPath, followed by pixel classification in ilastik to detect true signals. The SAM API refines object borders, and ROIs are saved for macro processing. (B) This method measures collagen α5(IV) levels in BMs of isogenic control, severe, and moderate models before and after ASO treatment. Fluorescent channels (one per protein) are separated. The channel of interest is imported into ilastik for random-forest–based pixel classification. Using the QuPath and ilastik project files, the macro scans the raw images and performs quantification, generating CSVs with full border area, mean channel intensity at the border and inside the object, and channel area. Protein quantity in tubular BM (TBM) and glomerular BM (PEC-BM) is calculated as mean border intensity × border area; GBM quantity as mean intraglomerular intensity × intraglomerular area.

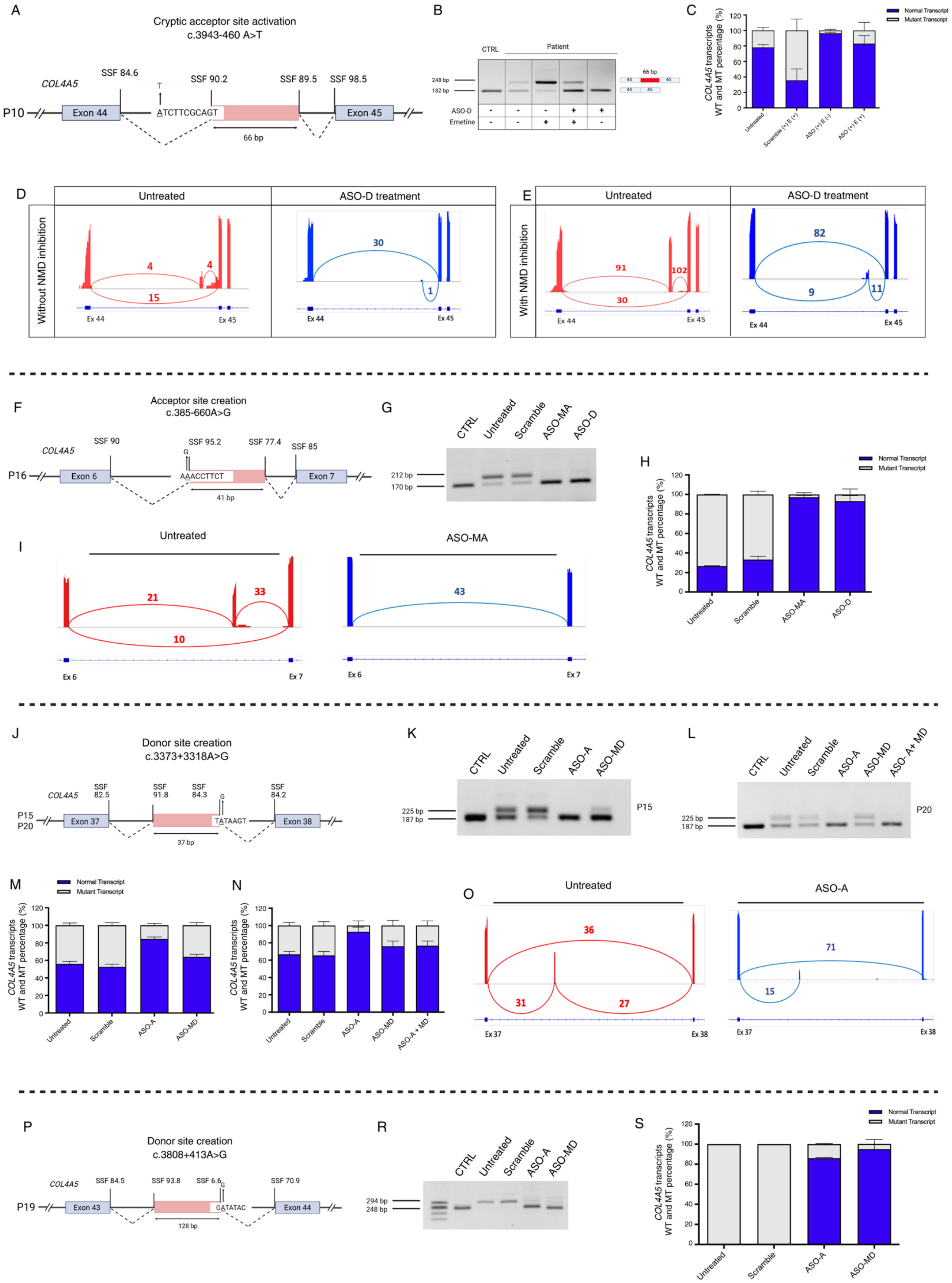

A
Cryptic acceptor site activation
c.3943-460 A>T
COL4A5
SSF 84.6
SSF 90.2
SSF 89.5
SSF 98.5
T
P10
Exon 44
ATCTTCGCAGT
66 bp
Exon 45
B
CTRL
Patient
248 bp
182 bp
66 bp
44
45
ASO-D
Emetine
C
COL4A5 transcripts WT and MT percentage (%)
Normal Transcript
Mutant Transcript
Untreated
Scramble (+) E (+)
ASO (+) E (-)
ASO (+) E (+)
D
Untreated
ASO-D treatment
Without NMD inhibition
4
4
15
30
1
Ex 44
Ex 45
E
Untreated
ASO-D treatment
With NMD inhibition
91
102
30
82
9
11
Ex 44
Ex 45
F
Acceptor site creation
c.385-660A>G
COL4A5
SSF 90
SSF 95.2
SSF 77.4
SSF 85
G
P16
Exon 6
AAACCTTCT
41 bp
Exon 7
G
CTRL
Untreated
Scramble
ASO-MA
ASO-D
212 bp
170 bp
H
Normal Transcript
Mutant Transcript
COL4A5 transcripts WT and MT percentage (%)
Untreated
Scramble
ASO-MA
ASO-D
I
Untreated
ASO-MA
21
33
10
43
Ex 6
Ex 7
J
Donor site creation
c.3373+3318A>G
COL4A5
SSF 82.5
SSF 91.8
SSF 84.3
SSF 84.2
G
P15
P20
Exon 37
TATAAGT
37 bp
Exon 38
K
CTRL
Untreated
Scramble
ASO-A
ASO-MD
225 bp
187 bp
P15
L
CTRL
Untreated
Scramble
ASO-A
ASO-MD
ASO- A+ MD
225 bp
187 bp
P20
M
Normal Transcript
Mutant Transcript
COL4A5 transcripts WT and MT percentage (%)
Untreated
Scramble
ASO-A
ASO-MD
N
COL4A5 transcripts WT and MT percentage (%)
Untreated
Scramble
ASO-A
ASO-MD
ASO-A + MD
O
Untreated
ASO-A
36
31
27
71
15
Ex 37
Ex 38
P
Donor site creation
c.3808+413A>G
COL4A5
SSF 84.5
SSF 93.8
SSF 6.6
SSF 70.9
G
P19
Exon 43
GATATAC
128 bp
Exon 44
R
CTRL
Untreated
Scramble
ASO-A
ASO-MD
294 bp
248 bp
S
Normal Transcript
Mutant Transcript
COL4A5 transcripts WT and MT percentage (%)
Untreated
Scramble
ASO-A
ASO-MD

Supplemental Figure 5. Antisense oligonucleotides targeting cryptic splice sites in multiple *COL4A5* deep-intronic variants showed efficient splicing correction. (A) P10 variant activated cryptic acceptor site at intron 44. (B) RT-PCR results confirmed aberrant splicing in the patient cells with NMD-mediated mRNA degradation, with ASO-D treatment restoring normal splicing. (C) Quantification of wild-type and mutant transcripts confirmed splice correction after ASO treatment. Panels (D) and (E) showing splice junction usage with and without NMD inhibition, and successful pseudo-exon skipping after ASO treatment. (F) P16 variant created novel acceptor site resulting in 41bp retention of intron 6 sequence in the *COL4A5* mRNA (G). This aberrant splicing event was successfully corrected following ASO-MA and ASO-D treatment. Panels (H) and (I) showed the quantification of *COL4A5* transcripts and splice junction usage before and after ASO treatment. (J) In P15 and P20 the variant created a novel donor site, and RT-PCR analysis **(K, L)** showed the retention of 37bp from intron 37 which was effectively restored by ASO-A treatment. This was further validated by fragment analysis (M, and N) and targeted RNA-seq (O). (P) Details of a donor site creation in P19. RT-PCR analysis (R) demonstrated the restoration of normal transcript levels following ASO-A and ASO-MD treatment. (S) The effects of ASO treatments on transcript levels~~,~~ were further quantified by fragment analysis. The results of the fragment analyzer were analyzed using two-way ANOVA followed by Tukey’s multiple comparisons test.

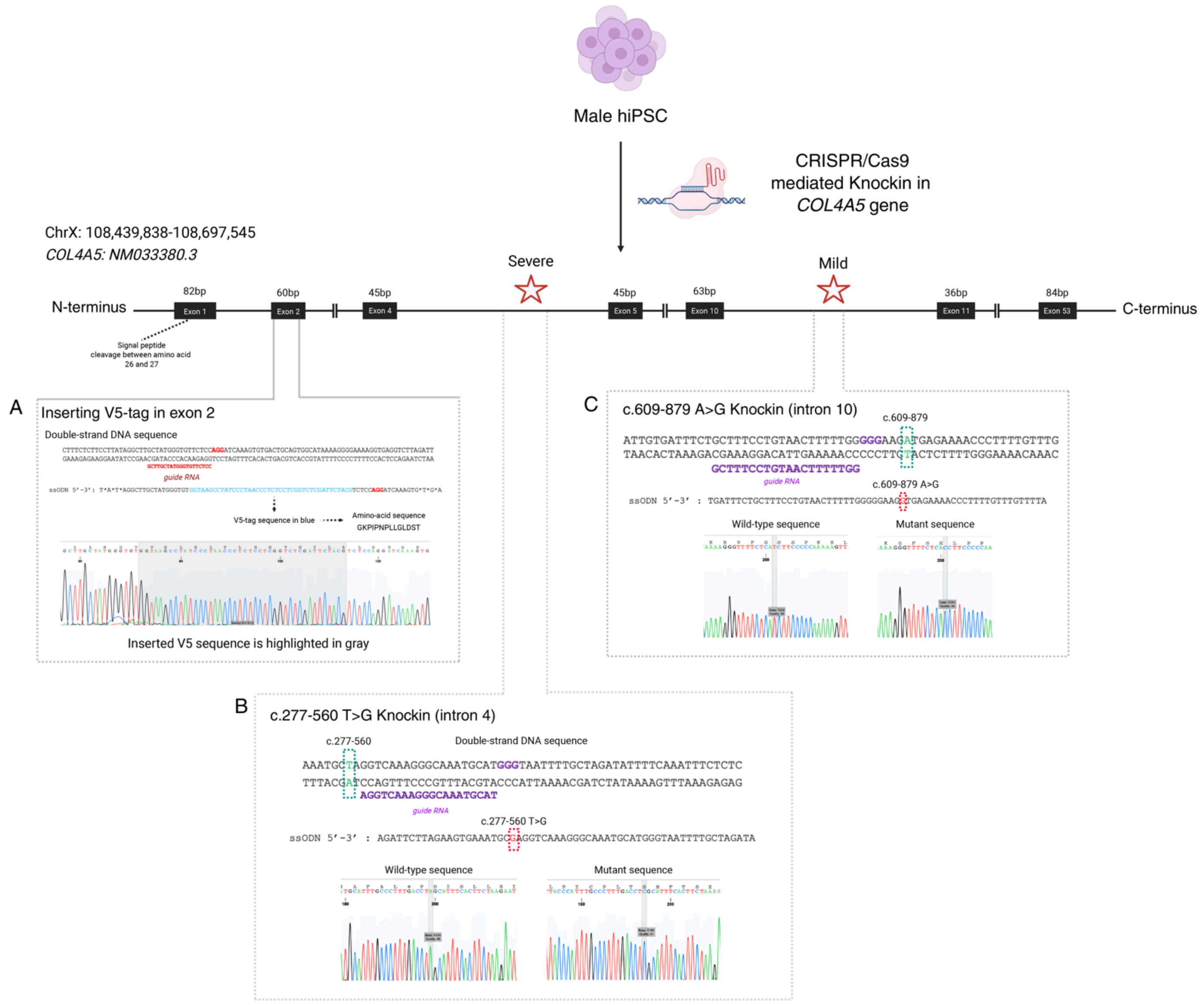

Supplemental Figure 6. Generation of isogenic hiPSC lines with CRISPR/Cas9-mediated knock-in of *COL4A5* variants. The figure illustrates the CRISPR/Cas9 strategy used to introduce V5-tag (A), severe (B) and mild (C) *COL4A5* variants in male hiPSCs for modeling X-linked Alport Syndrome. The top panel provides an overview of the *COL4A5* gene structure, highlighting the locations of the introduced variants. The left inset details the V5-tag knock-in strategy in exon 2, showing the insertion site (gray highlighted sequence) with Sanger sequencing confirming successful integration. The bottom-left inset depicts the severe c.277-560T>G variant knock-in in intron 4, showing the guide RNA (purple) and donor ssODN used for editing, along with Sanger sequencing verifying the mutation. The right inset outlines the mild c.609+879A>G variant knock-in in intron 10, presenting the guide RNA (purple), donor ssODN, and corresponding Sanger sequencing chromatograms confirming the mutant sequence. The generated clones were checked for karyotype and genome instability using SNP array.

Supplemental Table 8. Guide RNAs and ssODNs used for CRISPR/Cas9 mediated knock-ins.

Variant	Patient ID	Gene location	Guide RNA sequence	ssODN (sequence)	length
**c.277-560 T>G**	P3	*COL4A5* Intron 4	AGGTCAAAGGGCAAATGCAT	A*G*A*TTCTTAGAAGTGAAATGCGAGGTCAAAGGGCAA ATGCATGGGTAATTTTGCTAG*A*T*A	60nt
**c.609-879 A>G**	P14	*COL4A5* Intron 10	GCTTTCCTGTAACTTTTTGG	T*G*A*TTTCTGCTTTCCTGTAACTTTTTGGGGGAAGGTG AGAAAACCCTTTTGTTTGTT*T*T*A	60nt
**COL4A5-v5 tag**	-	*COL4A5* Exon 2	GCTTGCTATGGGTGTTCTCC	T*A*T*AGG CTT GCT ATG GGT GTG GTA AGC CTA TCC CTA ACC CTC TCC TCG GTC TCG ATT CTA CGT CTC CAG GAT CAA AGT G*T*G*A	82nt

*Phosphorothioate modifications

Supplemental Table 9. List of the antibodies used in this study.

No	Reagent type	Designation	Provider	Identifier	Additional comments
1	Antibody	Anti-laminin S/laminin β2 chain, clone CL2979 (mouse monoclonal)	Novus Biologicals	Cat# NBP-42387	IF (1:100)
2	Antibody	Anti-human collagen IV α3 chain NC1 domain, clone H31 (rat monoclonal)	Chondrex	Cat# 7076	IF (1:100)
3	Antibody	Anti-human collagen IV α4 chain NC1 domain, clone H43 (rat monoclonal)	Chondrex	Cat# 7073	IF (1:100)
4	Antibody	Anti-human collagen IV α6 chain NC1 domain, clone H63 (rat monoclonal)	Chondrex	Cat# 7074	IF (1:100)
5	Antibody	Anti-human collagen IV α5 chain, clone H53 and NC1, clone H52	Chondrex	Cat# 7078<br>Cat# 7077	IF (1:100)
6	Antibody	V5-Tag antibody I SV5-Pk1, mouse	Bio-rad	Cat# MCA1360	IF (1:100)
7	Antibody	Rabbit Anti-Claudin-1 (CLDN1), Rabbit Polyclonal Antibody	Bicell	Cat# 00201	IF (1:100)
8	Antibody	Human GATA-3 Antibody, Goat	R&D systems	Cat# AF2605	IF (1:100)
9	Antibody	Lotus Tetragonolobus Lectin (LTL), Biotinylated (B-1325-2)	Vector	Cat# B-1325-2	IF (1:50)
10	Antibody	Anti-laminin alpha 5/LAMA5, Mouse	Abcam	Ab210957	IF (1:100)
11	Antibody	Anti-laminin beta 1/LAMB1, Mouse	Merk	MAB1921P	IF (1/200)
12	Antibody	E-Cadherin (ECCD), Rat	Life technologies	Cat# B-1900	IF (1/400)
13	Antibody	Antibody anti-WT1, Mouse (F-6)	Santa Cruz	Cat# sc-7385	IF (1/100)
14	Antibody	Anti-human collagen IV, Rabbit	Rockland	Cat# 600-401-106-0.1	IF (1/200)
15	Antibody	Anti-human Nephrin, Sheep	R&D systems	Cat# AF4269	IF (1/100)
16	Antibody	Anti-human podocalyxin, Goat	R&D systems	Cat# AF1658	IF (1/500)
17	Antibody	Anti-human ZO-1/TJP1, Rabbit	Invitrogen	Cat# 40-2200	IF (1/100)
18	Antibody	Anti-human COL4A1-COL4A2	Developmental Studies Hybridoma Bank (DSHB)	Cat# M3F7-s	IF (1/100)
19	Antibody	PDGF Receptor B (28E1)	Cell Signaling	Cat# 3169	IF (1/400)
20	Antibody	Anti-α-Smooth Muscle Actin Antibody (ACTA2)	Sigma	Cat# A2547	IF (1/500)
21	Antibody	Collagen Type I Monoclonal antibody	ProteinTech	Cat# 67288-1-Ig	IF (1/400)
22	Antibody	HOPX Polyclonal antibody	ProteinTech	Cat# 11419-1-AP	IF (1/100)
23	Antibody	HMGB2 Polyclonal antibody	ProteinTech	Cat# 14597-1-AP	IF (1/100)

Supplemental Table 10. Primers used for RT-PCR and RT-qPCR.

Case	Primer	Sequence (5'-3')	Modification	length	Tm	GC	Transcript type	Product size
P3	Forward primer	CGGGGACAAAAGGGTGATGA	FAM	20	59.96	55	Wild type	177 bp
	Reverse primer	TCCACGTTCTCCCTTGGTTC	-	20	59.6	55	Mutant	235 bp
P14	Forward primer	CCTGGTCCCACTGGTATACC	FAM	20	58.87	60	Wild type	123 bp
	Reverse primer	GGGTCCCTGGAAATTTAAGCC	-	21	58.89	52	Mutant	251 bp
P10	Forward primer	GAAAGGAGATCAAGGACCACC	FAM	21	58.01	52.38	Wild type	182 bp
	Reverse primer	GAGGACCTGGAGGACCAATAA	-	21	58.52	52.38	Mutant	248 bp
P16	Forward primer	CACCAGGACCAAAAGGAATCA	FAM	21	58.12	47.62	Wild type	170 bp
	Reverse primer	ACCAGGAAAACCGGGACTG	-	19	59.55	57.89	Mutant	212 bp
P15 & P20	Forward primer	TGGTATCAAAGGTTCTGTGGGA	FAM	22	59.02	45.45	Wild type	187 bp
	Reverse primer	ATGACCTCCACCACCTACAG	-	20	58.43	55	Mutant	225 bp
P19	Forward primer	GAAGGACCTAAAGGCAACCCT	FAM	21	59.65	52.38	Wild type	284bp
	Reverse primer	CCTTTCATGCCTGGGAATCC	-	20	58.59	55	Mutant	330bp
COL4A1	Forward primer	ACTACTCGTACTGGCTGTCC	-	20	58.54	55	Product size	151bp
	Reverse primer	GGGATCTGAATGGTCTGGCT	-	20	59.16	55		
COL4A2	Forward primer	CCAGGACAGAAAGGAGACCA	-	20	58.65	55	Product size	112bp
	Reverse primer	CAGGTGCCCCAGAGATGTT	-	19	59.31	57.89		
COL4A3	Forward primer	GTCAACACCAGCTCTGATGC	-	20	59.2	55	Product size	167bp
	Reverse primer	TCCAGAGAGAAATCCAGCCG	-	20	59.17	55		
COL4A4	Forward primer	CAGGCATGAGAGGACCAGAA	-	20	59.09	55	Product size	162bp
	Reverse primer	CGTCTTCTCCTGTGTCACCT	-	20	59.04	55		
COL4A5	Forward primer	CAGACGATCCAGATTCCCCATTGT	-	24	62.50	50	Product size	122bp
	Reverse primer	GAACCAGGGGAGGCTAGGGCTTGA	-	24	62.50	62		
HPRT1	Forward primer	TCTTTGCTGACCTGCTGGATT	-	21	59.93	47.62	Product size	151bp
	Reverse primer	GTTGAGAGATCATCTCCACCAATTACT	-	27	60.63	40.74		
